# Genome-wide cross-trait analysis to compare genetic architecture between Parkinson’s disease and kidney-related traits

**DOI:** 10.1038/s41467-026-77920-3

**Published:** 2026-09-18

**Authors:** Le Chang, Sadaf Gawhary, Lyza Maameri, Wiame Belbellaj, Frida Lona-Durazo, Sarah A. Gagliano Taliun

**Affiliations:** 1 Faculty of Medicine, Université de Montréal, Montréal, QC Canada; 2 Research Centre, Montréal Heart Institute, Montréal, QC Canada; 3 Faculty of Medicine, Université Laval, Québec, QC Canada; 4 Department of Medicine, Faculty of Medicine, Université de Montréal, Montréal, QC Canada; 5 Department of Neurosciences, Faculty of Medicine, Université de Montréal, Montréal, QC Canada

**Keywords:** Medical genetics, Genetics research, Genome-wide association studies

## Abstract

Epidemiological studies link kidney function to Parkinson’s disease risk, but the genetic basis of this relationship remains unclear. Here, we leverage genome-wide association data for Parkinson’s disease and five UK Biobank-derived kidney-related traits in individuals of European ancestry, combining genetic correlation, pleiotropy mapping, colocalization and enrichment across sex-combined and sex-stratified analyses. Genome-wide genetic correlations with Parkinson’s are modest, with only a significant correlation with urinary potassium-to-creatinine ratio significant, yet we identify shared loci across 9 of 15 trait pairs. Resolving these loci, the two traits are more often driven by different causal variants within a shared region than by the same variant. Four loci carry a shared causal variant, implicating *BIN3*, *MSRA* and *TNK2* in protein quality control. These findings provide evidence for shared genomic loci between Parkinson’s disease and kidney function, offering specific loci and biological pathways for future investigation of the kidney-brain axis in neurodegeneration.

## Abstract (web summary)

Parkinson’s disease has been epidemiologically linked to kidney function, but the extent of their shared genetic basis remains unclear. Here, the authors integrate genome-wide genetic correlation, pleiotropy and colocalisation analyses to identify shared loci and candidate pathways connecting Parkinson’s disease and kidney-related traits.

## Introduction

Parkinson’s disease (PD) is the fastest-growing neurodegenerative disease worldwide, affecting over 8.5 million individuals and imposing a substantial burden on patients, caregivers, and healthcare systems^1,2^. Emerging evidence suggests that PD risk and progression may be influenced by organ systems beyond the brain, including the kidneys. For instance, a retrospective cohort study found a 1.55-fold higher incidence of PD among individuals with end-stage renal disease compared to matched controls^3^, and a population-based longitudinal study reported that individuals with chronic kidney disease (CKD) aged 55 years and older developed PD more frequently during a 10-year follow-up period^4^. Additionally, CKD and PD share several risk factors, including diabetes, hypertension, vitamin D deficiency, and anemia^5^. Therefore, understanding the potential mechanisms underlying the kidney-PD connection could reveal avenues for risk stratification and intervention.

Multiple pathophysiological pathways have been hypothesized to link kidney health and neurodegeneration. CKD leads to accumulation of uremic toxins, which promote oxidative stress and systemic inflammation processes that impair mitochondrial function and contribute to neuronal damage^6^. Vascular dysfunction, including hypertension-driven microvascular injury, can simultaneously damage both the renal and cerebral microvasculature through a shared strain vessel mechanism^7^. Furthermore, the kidneys and the brain express components of the renin-angiotensin system, and dysregulation of this system has been implicated in dopaminergic neurodegeneration in PD^8^. While these mechanistic links are plausible, the evidence from epidemiological and causal inference studies remains mixed, and the genetic underpinnings potentially influencing both kidney health and PD have not been thoroughly investigated^6,9^.

Genome-wide association studies (GWAS) have identified numerous risk loci for both PD and kidney-related traits, such as estimated glomerular filtration rate (eGFR), revealing substantial heritability for both conditions^10–12^. Cross-trait genetic analysis is a powerful strategy for detecting shared genetic signals between complex diseases and traits, as demonstrated by recent large-scale studies characterizing pleiotropy across organ systems^13,14^. However, this approach has not been applied to the kidney-PD axis, and the question of whether kidney function and PD share specific genetic loci and biological pathways remains underexplored.

**Fig. 1:**
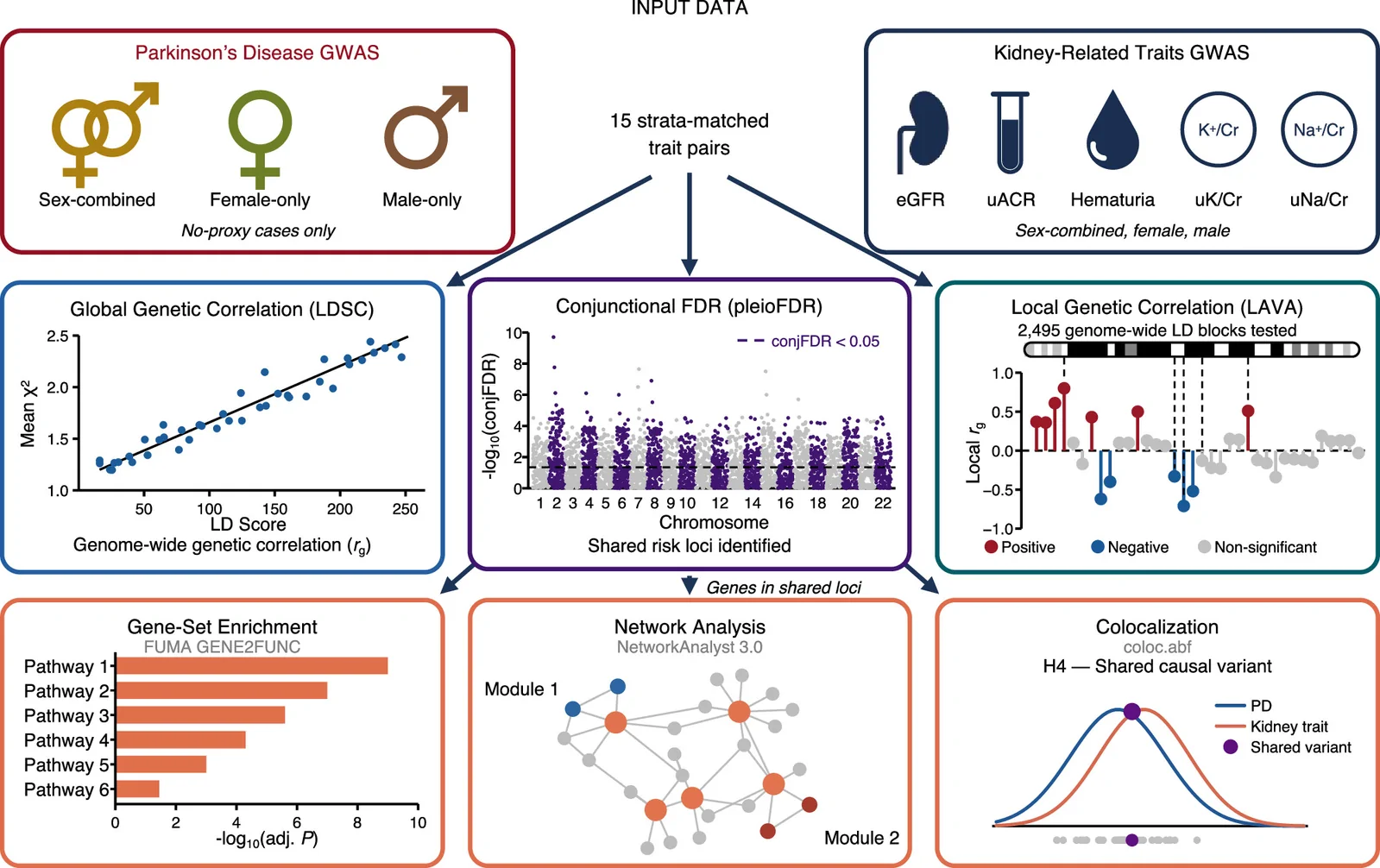
Analytical workflow for assessing the genetic relationship between Parkinson’s disease and kidney-related traits. Genome-wide association study (GWAS) summary statistics were obtained for Parkinson’s disease (PD) and five kidney-related traits: estimated glomerular filtration rate (eGFR), urinary albumin-to-creatinine ratio (uACR), hematuria, urinary potassium-to-creatinine ratio (uK/Cr), and urinary sodium-to-creatinine ratio (uNa/Cr). PD summary statistics included sex-combined meta-analyzed, female-only, and male-only strata (excluding proxy cases). Each kidney trait was similarly available in sex-combined, female-only, and male-only strata, yielding 15 PD-kidney trait pairs. These pairs were analyzed using three complementary approaches. First, cross-trait linkage disequilibrium (LD) score regression (LDSC) estimated genome-wide genetic correlations (*r*_g_) between each trait pair. Second, conjunctional false discovery rate (conjFDR) analysis, implemented in the pleioFDR framework, identified specific genomic loci harboring variants jointly associated with both PD and kidney traits at conjFDR <0.05. Third, Local Analysis of [co]Variant Association (LAVA) tested for local genetic correlations across 2,495 approximately independent LD blocks spanning the genome, identifying regions with significant positive or negative local genetic sharing. Downstream analyses were performed on loci emerging from the conjFDR results. Protein-coding genes mapped to shared conjFDR loci were evaluated for enrichment in biological pathways using FUMA GENE2FUNC and for protein–protein interaction (PPI) network modules using NetworkAnalyst 3.0. Shared loci were further assessed using Bayesian colocalization (coloc.abf) to distinguish shared from distinct causal variants. Urinary biomarkers were dilution-corrected by normalization to urinary creatinine. All summary statistics were derived from individuals of European genetic ancestry and aligned to genome build GRCh37 (hg19). This schematic was created by the authors using Microsoft PowerPoint; it contains no previously published or third-party material.

In this study, we leverage large-scale GWAS summary statistics for PD and five kidney-related traits—eGFR, urinary albumin-to-creatinine ratio (uACR), hematuria, urinary potassium-to-creatinine ratio (uK/Cr), and urinary sodium-to-creatinine ratio (uNa/Cr)—to perform a systematic cross-trait analysis using computational genetics approaches to identify shared loci (Fig. 1). We evaluate genome-wide and local genetic correlations using cross-trait linkage disequilibrium (LD) score regression (LDSC) and Local Analysis of (co)Variant Association (LAVA). We also identify specific shared loci using conjunctional false discovery rate (conjFDR) analysis, followed by Bayesian colocalization to distinguish shared from distinct causal variants and functional enrichment analyses to characterize the biological pathways implicated. All analyses are conducted across sex-combined and sex-stratified strata using PD GWAS excluding proxy cases, in individuals of European genetic ancestry.

## Results

### Global genetic correlations

We estimated genome-wide genetic correlations (*r*_g_) between PD and five kidney-related traits using LDSC across 15 trait pairs (Supplementary Data 1, 2). LDSC intercepts were close to 1 across all traits (range: 0.90–1.08), indicating minimal confounding from population stratification.

**Fig. 2:**
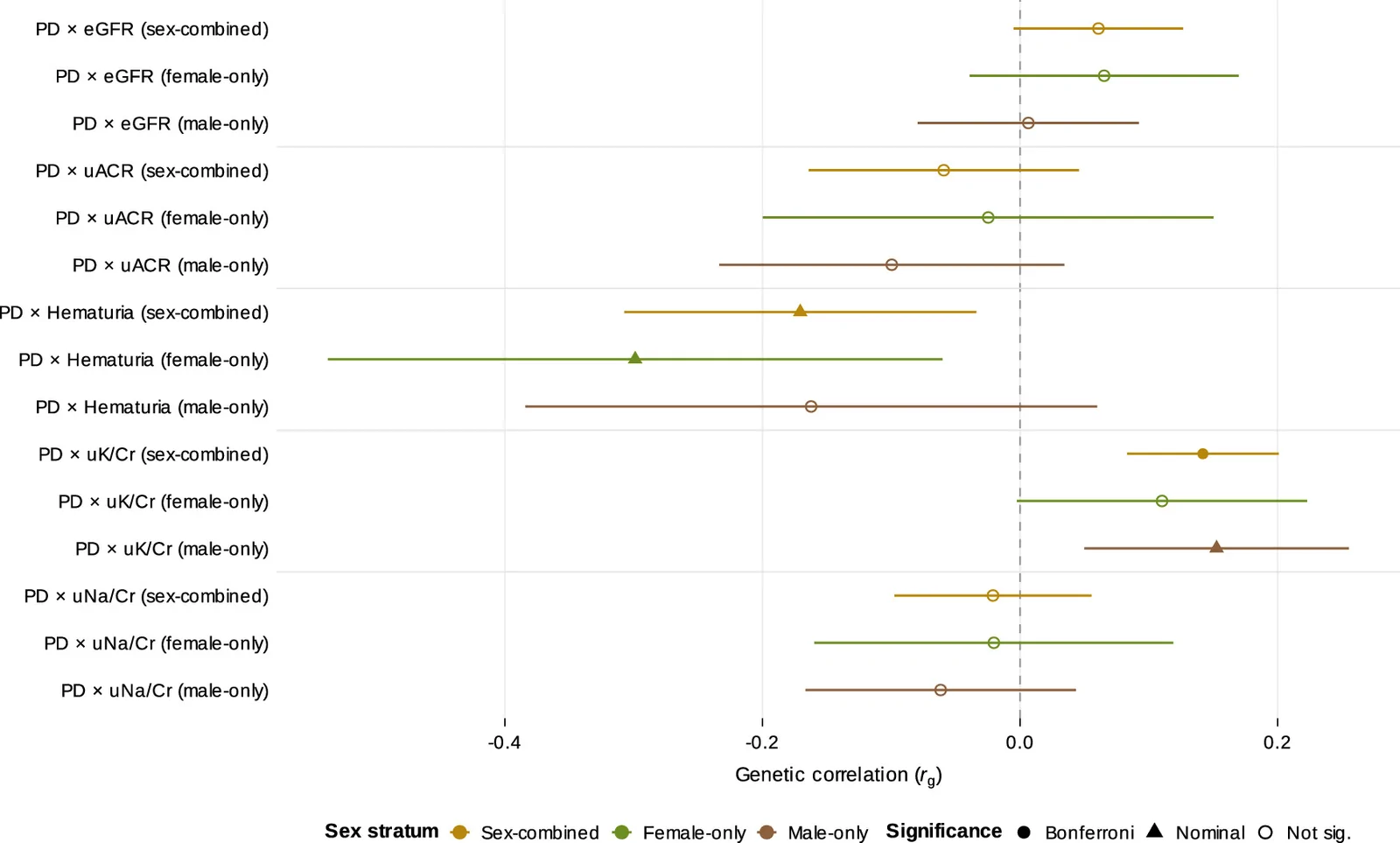
Genome-wide genetic correlations between Parkinson’s disease and kidney-related traits estimated by cross-trait linkage disequilibrium (LD) score regression. Forest plot showing genetic correlation estimates (*r*_g_) and 95% confidence intervals for each of the 15 Parkinson’s disease (PD)–kidney trait pairs. Trait pairs are grouped by kidney phenotype: estimated glomerular filtration rate (eGFR), urinary albumin-to-creatinine ratio (uACR), hematuria, urinary potassium-to-creatinine ratio (uK/Cr), and urinary sodium-to-creatinine ratio (uNa/Cr). Each pair was analyzed across three sex strata: sex-combined (gold), female-only (olive green), and male-only (brown). Each estimate derives from two genome-wide association studies; sample sizes for each trait and sex stratum are listed in Table 1 (PD: *n* = 60,633, 28,277 and 32,356 individuals for the sex-combined, female-only and male-only strata, respectively; kidney traits: *n* = 59,199 to 1,201,930 individuals). Points denote the genetic correlation point estimate, which is the measure of center, and horizontal lines the corresponding 95% confidence interval (estimate ± 1.96 × the LD score regression standard error). The vertical dashed line indicates *r*_g_ = 0 (no genetic correlation). *P* values are two-sided and were derived from the LD score regression Z statistic; the multiple-comparison adjustment is a Bonferroni correction for the 15 trait pairs tested and is shown by symbol shape: filled circles indicate Bonferroni-corrected significance (*P* < 0.05/15 = 3.33 × 10⁻³), filled triangles nominal significance (*P* < 0.05), and open circles non-significant results. Positive *r*_g_ values indicate sha*r*ed genetic risk (variants that increase PD risk also increase the kidney trait), while negative values indicate opposing genetic effects. PD × uK/Cr in the sex-combined stratum survived Bonferroni correction, and several pairs involving hematuria and uK/Cr reached nominal significance. All analyses used summary statistics from individuals of European genetic ancestry aligned to genome build GRCh37 (hg19). Source data are provided as a Source Data file.

The most significant genetic correlation was between PD-uK/Cr in the sex-combined analysis (*r*_g_ = 0.14, standard error (SE) = 0.03, *P* = 2.2 × 10⁻⁶), the only pair surpassing Bonferroni correction (*P* < 3.3 × 10⁻³). This positive correlation was consistent in male-specific (*r*_g_ = 0.15, *P* = 3.6 × 10⁻³) and directionally concordant in female-specific (*r*_g_ = 0.11, *P* = 0.055) analyses. Nominally significant negative correlations were observed between PD-hematuria in the sex-combined (*r*_g_ = −0.17, *P* = 0.014) and female-specific (*r*_g_ = −0.30, *P* = 0.014) analyses. PD-eGFR showed a trend toward positive correlation (*r*_g_ = 0.06, *P* = 0.07). No significant correlations were observed for PD-uACR or PD-uNa/Cr (Fig. 2).

### Identification of shared genomic loci between PD and kidney traits

To identify specific loci jointly associated with PD and kidney traits, we applied conjFDR analysis, which leverages cross-trait enrichment to detect shared genetic signals that may not reach genome-wide significance in single-trait analyses. No LAVA results passed multiple testing correction (Supplementary Data 17). Full conjFDR results are provided in Supplementary Data 3–11 and Supplementary Fig. 1.

At conjFDR <0.05, we identified shared loci in 9 of the 15 tested trait pairs. PD-eGFR (sex-combined) yielded the most extensive signal (61 distinct shared loci). Threshold sensitivity is reported in Supplementary Note 4 and Supplementary Data 19. The most significant signals included *FAM47E-STBD1* on chromosome 4 (lead single nucleotide polymorphism (SNP) rs6812193, conjFDR = 2.6 × 10⁻⁷), a region harboring the established PD risk gene *SCARB2*; *HLA-DRB1* in the major histocompatibility complex (MHC) region (rs7760841, conjFDR = 5.9 × 10⁻⁷); *CTSB* on chromosome 8 (rs1736103, conjFDR = 2.6 × 10⁻⁶); and *TMEM163* on chromosome 2 (rs6707326, conjFDR = 4.6 × 10⁻⁶). Effect directions were mixed across loci: at some, PD risk alleles were associated with higher eGFR (e.g., rs6707326 near *TMEM163*, rs4242434 near *BIN3*), while at others the relationship was inverted (e.g., rs6812193 near *FAM47E-STBD1*, rs12667917 near *RAB19*). In the remaining sex-combined analyses, PD-uNa/Cr yielded the next most shared loci (11), followed by PD-uACR (5), PD-uK/Cr (4), and PD-hematuria (1).

Sex-stratified PD-eGFR analyses identified 15 female-specific and 12 male-specific shared loci. Core loci—*FAM47E-STBD1/SCARB2*, *CTSB*, and *TMEM163*—recurred across strata, while *TNK2* and *ZZZ3* were identified only in the female-specific analysis, and *RAB19* only in male-specific and sex-combined analyses. Several loci also recurred across trait pairs: *FAM47E-STBD1* appeared in four pairs, while *FGFRL1*, *CTSB* and *GTF3C2* each appeared in three.

### Colocalization and eQTL annotation

To determine whether PD and kidney traits shared the same causal variant at identified conjFDR loci, we performed Bayesian colocalization (coloc.abf) at each non-MHC locus across the seven trait pairs (128 locus-trait-pair tests spanning 70 distinct loci; Supplementary Data 12). Distinct causal variants were nearly three times more common than shared ones: 11 of the 70 loci showed evidence for distinct causal variants (PP.H3 > 0.80), including the PD risk locus *FAM47E-STBD1/SCARB2* (PP.H3 = 0.9999), whereas four loci demonstrated evidence for a shared causal variant (PP.H4 > 0.80). Altogether, these results indicate that while PD and kidney-function signals frequently converge at the same genomic regions, they are typically driven by distinct causal variants. These colocalization calls held across the grid of priors tested down to *p*_12_ = 5 × 10^−6^; at the most stringent setting (*p*_12_ = 1 × 10^−6^) four of the five shared-variant calls fell below the 0.80 threshold, although PP.H4 continued to exceed PP.H3 at every locus (Supplementary Note 2 and Supplementary Data 20). None of the calls were flagged for meta-analysis fine-mapping miscalibration by the SLALOM diagnostic (Supplementary Note 3 and Supplementary Data 21)^15^.

**Fig. 3:**
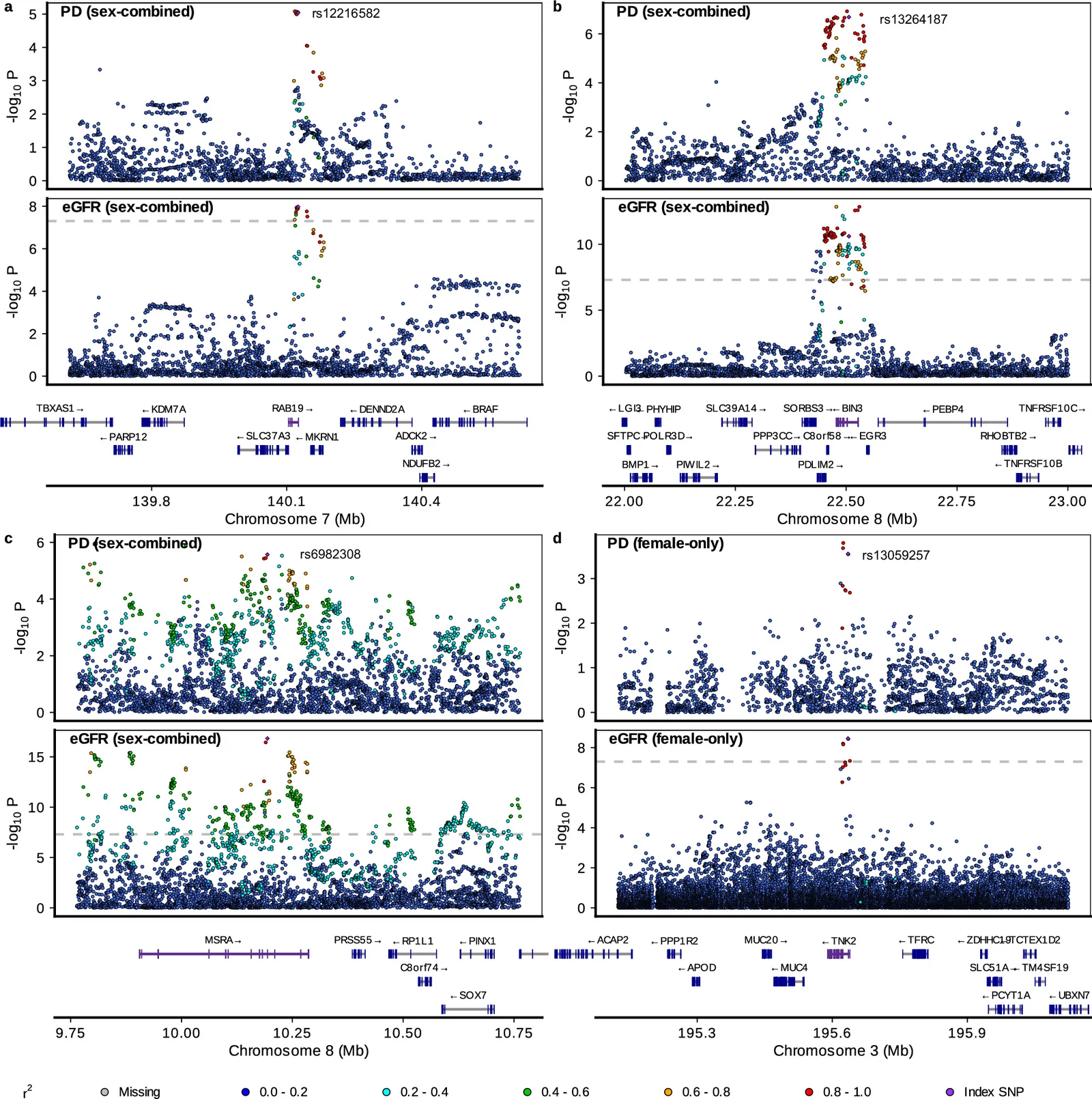
Regional association plots at four colocalized loci between Parkinson’s disease and estimated glomerular filtration rate. **a** Chromosome 7 near *RAB19* (rs12216582, PP.H4 = 0.96, sex-combined). **b** Chromosome 8 near *BIN3* (rs13264187, PP.H4 = 0.87, sex-combined). **c** Chromosome 8 near *MSRA/PRSS51* (rs6982308, PP.H4 = 0.93, sex-combined). **d** Chromosome 3 near *TNK2* (rs13059257, PP.H4 = 0.83, female-only). Each locus shows evidence for a shared causal variant, identified through conjunctional false discovery rate (conjFDR) analysis followed by Bayesian colocalization (coloc.abf). ConjFDR values are two-sided and constitute the multiple-comparison adjustment; loci were declared shared at conjFDR <0.05. Colocalization is a Bayesian model comparison rather than a null hypothesis test: it estimates five mutually exclusive posterior probabilities (PP.H0–PP.H4) for no association (H0), association with Parkinson’s disease (PD) only (H1), with the kidney trait only (H2), independent causal variants (H3), or a single shared causal variant (H4); no further multiple-comparison adjustment applies. Loci shown met stringent criteria (PP.H4 ≥ 0.8) at priors *p*_1_ = *p*_2_ = 1 × 10⁻⁴ and *p*_12_ = 5 × 10⁻⁶. Within each panel, the PD association signal is plotted directly above the estimated glomerular filtration rate (eGFR) signal for the same genomic window (±500 kb from the shared causal variant). The y-axis shows −log_10_P from the source genome-wide association studies (two-sided) and the x-axis shows genomic position in megabases (Mb); sample sizes are given in Table 1. Each point is a single nucleotide polymorphism (SNP), colored by linkage disequilibrium (r²) with the candidate shared causal variant, which is drawn as a purple diamond (1000 Genomes Phase 3 European reference panel); the same index SNP is used in both plots of a panel. SNPs without available r² data are shown in gray. The dashed horizontal line indicates genome-wide significance (*P* = 5 × 10⁻⁸). Gene annotations are shown beneath each plot. All colocalized loci are listed in Supplementary Data 12. All summary statistics derive from individuals of European genetic ancestry aligned to genome build GRCh37 (hg19). Source data are provided as a Source Data file.

All four shared-variant loci fell within PD-eGFR pairs: chromosome 7 near *RAB19* (PP.H4 = 0.96 sex-combined), chromosome 8 near *MSRA/PRSS51* (PP.H4 = 0.93 sex-combined) and near *BIN3* (PP.H4 = 0.87 sex-combined, 0.75 male-specific), and chromosome 3 near *TNK2* (PP.H4 = 0.83, female-specific) (Fig. 3). No trait pair outside PD-eGFR met the stringent threshold; although, several PD-uNa/Cr loci showed suggestive evidence, including a region near *CXCR4* (PP.H4 = 0.67 sex-combined).

To prioritize candidate effector genes, we annotated the candidate shared variant at each colocalized locus against cis-expression quantitative trait loci (cis-eQTLs) in brain tissues from the Genotype-Tissue Expression project (GTEx v11) and in the kidney glomerular and tubulointerstitial compartments (NephQTL2); the complete annotation is provided in Supplementary Data 13, 14. At three of the loci meeting stringent criteria for a shared causal variant, the candidate shared variant was a brain cis-eSNP for a candidate effector with evidence relevant to PD: *BIN3* (rs13264187) across the caudate, putamen, substantia nigra, hippocampus, and brain cortex; *MSRA* (rs6982308) in spinal cord; and *TNK2* (rs13059257), a female-specific signal, in brain cortex. On the kidney side, the chromosome 8 variant (rs13264187) was a significant cis-eSNP for podocyte-expressed *CCAR2* in both the glomerular and tubulointerstitial compartments. *BIN3*’s 95% credible set was broad (21 variants; top-posterior variant rs7005025), and within it rs13264187 was prioritized as the candidate effector variant by its brain and *CCAR2* cis-eQTL evidence and its recurrence as the lead variant in the male-specific analysis (Supplementary Data 12).

### Functional characterization of shared PD-kidney trait loci

To characterize the biological functions of genes within shared loci, we performed gene-set enrichment (FUMA GENE2FUNC) and protein-protein interaction (PPI) network enrichment analyses (NetworkAnalyst) on genes mapped to the conjFDR-identified loci for each PD-kidney trait pair (Supplementary Data 15, 16). PD-eGFR yielded the most extensive signal. The sex-combined PPI module was enriched for ubiquitin-mediated proteolysis (FDR-adjusted *P* = 2.0 × 10⁻³), cell-cycle regulation (FDR-adjusted *P* = 2.4 × 10⁻³), autophagy (FDR-adjusted *P* = 2.2 × 10⁻²) and HIF-1 signaling (FDR-adjusted *P* = 2.2 × 10⁻²), whereas the male-specific module was enriched for cellular senescence (FDR-adjusted *P* = 8.0 × 10⁻⁴) and longevity regulation (FDR-adjusted *P* = 0.0192). FUMA enrichment results implicated lipid and inflammatory biology: genes at PD-uNa/Cr and PD-uACR loci were enriched for hypertriglyceridaemia (FDR-adjusted *P* = 1.2 × 10⁻⁷) and inflammatory bowel disease (FDR-adjusted *P* = 0.0021). All analyses excluded the MHC region.

## Discussion

In this study, we performed a systematic cross-trait computational genetic analysis to characterize the genetic relationship between PD and five kidney-related traits. Although epidemiological studies consistently link chronic kidney disease to elevated PD risk, the genetic basis of this connection has remained undefined. The conjFDR analysis identified shared loci in 9 of the 15 tested trait pairs, with PD-eGFR yielding the most extensive signal (61 shared loci at conjFDR <0.05). We found, however, that genome-wide genetic correlations were modest — the strongest being between PD-uK/Cr (*r*_g_ = 0.14, *P* = 2.2 × 10⁻⁶). This pattern of weak global correlation alongside numerous discrete shared loci indicates that the genetic relationship between PD and kidney function may be driven by specific genomic regions rather than diffuse polygenic overlap, which could partially explain why prior global correlation and Mendelian randomization analyses in the literature have yielded inconsistent results.

Resolving whether the identified loci reflect the same or distinct causal variants helps interpret this genetic overlap. Bayesian colocalization revealed that distinct causal variants were nearly three times more common than shared ones at pleiotropic loci (PP.H3 > 0.80 at 11 of 70 distinct loci vs. PP.H4 > 0.80 at 4), including the PD risk locus *FAM47E-STBD1/SCARB2*, indicating that genetic signals for PD and kidney function frequently converge at the same genomic regions but through independent regulatory mechanisms. Four loci met the stringent threshold for a shared causal variant, all within PD-eGFR pairs. Among these, eQTL annotation in PD-relevant brain regions implicated candidate effector genes at three colocalized regions: *BIN3*, whose candidate shared variant (rs13264187) was a significant eQTL across the caudate, putamen, substantia nigra, hippocampus, and brain cortex; *MSRA* (rs6982308), with eQTL evidence in spinal cord; and *TNK2* (rs13059257), a female-specific colocalized signal with eQTL evidence in brain cortex. On the kidney side, the same variant (rs13264187) regulated a different gene: in both the glomerular and tubulointerstitial compartments, it was an eQTL for the neighboring *CCAR2*, not *BIN3*. *CCAR2*, expressed in podocytes, inhibits the sirtuin *SIRT1* and regulates cellular senescence^16^.

These variant-level findings were mirrored at the pathway level. Genes mapped to pleiotropic PD-eGFR loci were enriched for ubiquitin-mediated proteolysis, autophagy, cell-cycle regulation, and HIF-1 signaling in the sex-combined analysis, with male-specific enrichment for cellular senescence and longevity regulation. These pathways converge on proteostasis, a process central to PD pathogenesis through the accumulation of misfolded α-synuclein^17^ and equally implicated in kidney injury, where protein misfolding and ER stress drive glomerular and tubular damage^18^. That the same theme emerged independently from colocalization and enrichment analyses strengthens the inference: the eQTL-confirmed genes themselves are proteostasis effectors, with *BIN3* mediating endocytic trafficking, *MSRA* repairing oxidatively damaged proteins, and *MKRN1* targeting substrates for proteasomal degradation. The male-specific enrichment for cellular senescence is notable given the male predominance of PD^19^, and is corroborated at the variant level by the glomerular effector *CCAR2*: because it is expressed in podocytes, where senescence is a recognized driver of glomerular ageing and declining filtration^20^, this effector ties the senescence signal directly to the kidney. This aligns with evidence that cellular senescence contributes to both neuronal and renal aging^6^. In contrast, genes at PD-uNa/Cr and PD-uACR loci implicated lipid metabolism and inflammatory bowel disease pathways, suggesting that different kidney-related traits may capture distinct aspects of the kidney-brain connection, with eGFR reflecting proteostasis and protein quality control, while urinary biomarkers may capture systemic metabolic and inflammatory biology^6^.

Our study has several strengths. The study systematically distinguishes pleiotropic loci from shared causal variants between PD and multiple kidney-related traits using complementary approaches spanning global genetic correlation, locus-specific pleiotropy mapping, colocalization, and functional enrichment. The use of five kidney-related traits capturing distinct aspects of renal and urinary physiology, combined with sex-stratified analyses using PD GWAS excluding proxy cases, provides a comprehensive characterization of the PD-kidney genetic connection. The integration of colocalization with eQTL annotation enabled resolution of shared versus distinct causal signals and candidate functional gene assignment beyond positional proximity. Some limitations should be noted. First, all analyses were restricted to individuals of European genetic ancestry, and these findings may not generalize to other populations. Second, creatinine-based eGFR estimation may partially reflect muscle mass metabolism in addition to kidney function. We therefore conducted a sensitivity analysis using cystatin C-based eGFR (Supplementary Note 1 and Supplementary Data 18), which is largely independent of muscle mass and in which the primary shared locus (*BIN3*) was recovered (Supplementary Data 18, 19). Third, our findings describe genetic correlations and pleiotropic associations rather than causal relationships; previous Mendelian randomization studies examining kidney function and neurodegeneration have yielded largely null results^6^. Fourth, the enrichment analyses based on the prize-collecting Steiner forest algorithm include both seed genes from conjFDR loci and algorithmically inferred connector genes, and should therefore be interpreted as reflecting the broader network context of shared loci. Fifth, colocalization posteriors depend on *p*_12_, the prior probability that a variant is causal for both traits. Our shared-variant calls hold across the priors in common use, but only *RAB19* remains above 0.80 at *p*_12_ = 1 × 10^−6^. The evidence at *MSRA/PRSS51*, *BIN3* and *TNK2* is therefore conditional on standard prior settings, although at no prior does it favor a distinct causal variant. Finally, eQTL evidence was queried from GTEx bulk tissue data, where brain regions were well-powered (*n* = 180–290 per region), but kidney cortex had fewer samples (*n* ≈ 100), limiting our ability to detect kidney-specific regulatory effects at colocalized loci. Larger kidney eQTL datasets and single-cell resolution, as they become available, may reveal tissue- and cell-type-specific regulatory mechanisms underlying the shared genetic signals identified here.

These findings carry potential clinical and translational implications. The convergence of PD and eGFR on proteostasis-related loci suggests that therapies targeting protein quality control—such as autophagy enhancers or proteasome modulators currently in preclinical development for neurodegeneration—may warrant evaluation for renoprotective effects, and vice versa. More immediately, the identification of shared genetic variants between PD and kidney function supports the rationale for monitoring kidney health in PD patients and neurodegenerative risk in CKD populations, particularly given the epidemiological evidence linking these conditions. The presence of sex-differential colocalized loci in both female- and male-specific analyses further underscores the value of sex-stratified approaches in characterizing the kidney-brain axis.

In summary, our study identified widespread pleiotropy between PD and kidney-related traits at certain genomic loci, for which most were driven by a distinct causal variant. Functional enrichment analysis of genes within colocalized loci suggested proteostasis, oxidative stress repair, and membrane dynamics. eQTL annotation confirmed brain-relevant target genes at key colocalized loci, with *BIN3*, *MSRA*, and *TNK2* supported by expression evidence in PD-relevant brain regions. These results provide a genetic foundation for the kidney-brain axis in PD and highlight specific biological mechanisms—particularly protein quality control, vesicle trafficking, and cellular senescence—that may represent shared vulnerabilities across organ systems during aging.

## Methods

### Ethics

This study analyzed genome-wide association study summary statistics and individual-level UK Biobank data; no new participant data were collected. This research complies with all relevant ethical regulations. The study is a secondary analysis of genome-wide association study summary statistics and of individual-level data from the UK Biobank. The UK Biobank has ethical approval from the North West Multi-center Research Ethics Committee, and all UK Biobank participants provided written informed consent. Researchers can operate under this approval with no need for further ethical approval. The analyses reported here were carried out under UK Biobank application number 66222, and use of these data complied with the terms and conditions set by the UK Biobank. Summary statistics obtained from published studies were generated under the ethical approvals and participant consent reported in the corresponding primary publications. The design and conduct of this study complied with all relevant regulations governing the use of human study participants and was carried out in accordance with the criteria set by the Declaration of Helsinki.

### Sex and gender

Gender was not analyzed in the context of this study. The genome-wide association summary statistics used were either sex-combined or sex-specific.

### Genome-wide association study (GWAS) summary statistics

We collected sex-combined and sex-stratified genome-wide association study (GWAS) summary statistics with large sample sizes for PD as well as for five kidney-related traits (Table 1)^10,12,21,22^. Given prior findings that genetic correlation results diverge using GWAS results from a late-onset neurodegenerative disease (Alzheimer’s) that include proxy cases (i.e., individuals who do not have the disease of interest themselves, but who report having a parent with the disease)^23^, we carried out analyses in PD summary statistics without proxy cases. All summary statistics were derived from analyses carried out in individuals of European genetic ancestry. Expanded descriptions are provided in Supplementary Methods.

**Table 1:** Sources of genome-wide association study (GWAS) summary statistics

Trait	Sex stratum	Source (Reference)	Sample size	Phenotype definition
Parkinson’s disease	Sex-combined	Meta-analysis of Blauwendraat et al. 2021 (PMID: 33901317) sex-stratified data^12^	*n* = 60,633 (20,967 cases; 39,666 controls)	Clinically diagnosed PD cases without proxy cases. Controls aged >60 years with no PD or parental history of PD.
	Female-only	Blauwendraat et al. 2021, Ann Neurol (PMID: 33901317)^12^	*n* = 28,277 (7,947 cases; 20,330 controls)	Clinically diagnosed PD cases without proxy cases.
	Male-only	Blauwendraat et al. 2021, Ann Neurol (PMID: 33901317)^12^	*n* = 32,356 (13,020 cases; 19,336 controls)	Clinically diagnosed PD cases without proxy cases.
eGFR	Sex-combined	Stanzick et al. 2021, Nat Commun (PMID: 34272381) (CKDGen + UKB meta-analysis)^10^	*n* = 1,201,909	Creatinine-based eGFR estimated using the CKD-EPI 2009 equation. Meta-analysis of CKDGen Consortium (mostly European ancestry, *n* = 765,348) and UK Biobank (EUR, *n* = 436,581).
	Female-only	UK Biobank White British (internal analysis)	*n* = 209,516	Creatinine-based eGFR using CKD-EPI 2009 equation. Covariates: age, genetic PCs.
	Male-only	UK Biobank White British (internal analysis)	*n* = 178,711	Creatinine-based eGFR using CKD-EPI 2009 equation. Covariates: age, genetic PCs.
uACR	Sex-combined	UK Biobank White British (internal analysis)	*n* = 122,972	Urinary albumin-to-creatinine ratio (mg/g). Albumin values < 6.7 mg/L set to 6.7 (left-censored). Dilution-corrected. Covariates: age, sex, genetic PCs.
	Female-only	UK Biobank White British (internal analysis)	*n* = 59,199	As above. Covariates: age, genetic PCs.
	Male-only	UK Biobank White British (internal analysis)	*n* = 63,773	As above. Covariates: age, genetic PCs.
Hematuria	Sex-combined	Gagliano Taliun et al. 2022, CJASN (PMID: 35474271)^21^	*n* = 396,345 (16,409 cases; 379,936 controls)	ICD-code defined hematuria in UK Biobank White British participants. Sex-combined covariates: birth year, sex, genetic PCs. Sex-specific covariates: birth year, genetic PCs.
	Female-only	Lona-Durazo et al. 2026, Kidney Int Rep (PMID: 42433664)^22^	*n* = 220,094 (7,061 cases; 213,033 controls)	Same phenotype definition as sex-combined.
	Male-only	Lona-Durazo et al. 2026, Kidney Int Rep (PMID: 42433664)^22^	*n* = 187,440 (9,774 cases; 177,666 controls)	Same phenotype definition as sex-combined.
uK/Cr	Sex-combined	UK Biobank White British (internal analysis)	*n* = 394,601	Urinary potassium normalized to urinary creatinine (dilution-corrected). Covariates: age, sex, genetic PCs.
	Female-only	UK Biobank White British (internal analysis)	*n* = 212,733	As above. Covariates: age, genetic PCs.
	Male-only	UK Biobank White British (internal analysis)	*n* = 181,868	As above. Covariates: age, genetic PCs.
uNa/Cr	Sex-combined	UK Biobank White British (internal analysis)	*n* = 394,639	Urinary sodium normalized to urinary creatinine (dilution-corrected). Covariates: age, sex, genetic PCs.
	Female-only	UK Biobank White British (internal analysis)	*n* = 212,539	As above. Covariates: age, genetic PCs.
	Male-only	UK Biobank White British (internal analysis)	*n* = 182,099	As above. Covariates: age, genetic PCs.

*eGFR* estimated glomerular filtration rate, *uACR* urinary albumin-to-creatinine ratio, *uK/Cr* urinary potassium-to-creatinine ratio, *uNa/Cr* urinary sodium-to-creatinine ratio, *PCs* principal components, *CKD-EPI* Chronic Kidney Disease Epidemiology Collaboration, *UKB* UK Biobank, *EUR* European ancestry.

Urinary biomarkers (uACR, uK/Cr, uNa/Cr) were dilution-corrected by normalizing by urinary creatinine. All summary statistics were in GRCh37 and were restricted to individuals of European genetic ancestry. UK Biobank age at recruitment: 40–69 years.

In the sex-stratified PD GWAS, controls were participants with neither reported PD nor a parent with PD, and with an age of recruitment over 60 years old^12^. For the sex-combined PD GWAS, we eliminated the factor of proxy inclusion strategy, which varies depending on the study, by meta-analyzing the sex-stratified summary statistics together to derive sex-combined summary statistics. Specifically, using effect size estimates and their standard errors in METAL^24^, we meta-analyzed the PD female-specific dataset with the PD male-specific dataset without the inclusion of proxies to derive PD sex-combined summary statistics without proxies.

For kidney-related traits, we used five urinary and kidney function phenotypes (Table 1) carried out in individuals of European genetic ancestry, primarily in the UK Biobank, a large population-based cohort of individuals recruited across the UK, aged 40 to 69 years at recruitment. Sex-combined eGFR summary statistics were obtained from Stanzick et al.^10^, a meta-analysis of the CKDGen Consortium (median age of participants was 54 years^25^) and UK Biobank (*N* = 1,201,930). eGFR was estimated from serum creatinine using the CKD-EPI 2009 equation^26^. Sex-stratified eGFR GWAS were performed in UK Biobank White British participants (female *N* = 209,516; male *N* = 178,711), adjusting for age and genetic principal components. Hematuria was defined by ICD codes in UK Biobank White British participants (*N* = 396,345), as previously described^21^. The remaining urinary biomarkers—urinary albumin-to-creatinine ratio (uACR), urinary potassium-to-creatinine ratio (uK/Cr), and urinary sodium-to-creatinine ratio (uNa/Cr)—were derived from GWAS performed in UK Biobank White British participants (*N* = 122,972 to 394,639). Urinary electrolyte measurements (potassium and sodium) were normalized to urinary creatinine to correct for urine dilution, yielding dilution-corrected ratios (uK/Cr and uNa/Cr). Similarly, uACR is inherently dilution-corrected as a ratio of urinary albumin to urinary creatinine. Specifically, uACR values were computed as (albumin in mg/L)/(creatinine in μmol/L/1000) from urine samples collected at baseline. Individuals marked as having urinary albumin < 6.7 mg/L were assigned as having a urinary albumin value of 6.7 mg/L. All urinary biomarker GWAS were adjusted for age, sex (in sex-combined analyses), and genetic principal components. Full details of sample sizes, phenotype definitions, and data sources are provided in Table 1.

### Genetic correlation analyses

We computed pairwise genetic correlations between kidney traits and PD using bivariate (cross-trait) LD Score Regression (LDSC) v1.0.1^27^ for global correlations and Local Analysis of [co]Variant Association (LAVA) v0.1.5^28^ for local correlations^29,30^.

LDSC tests common autosomal variants using pre-computed LD scores based on the 1000 Genomes European ancestry super-population. We accounted for five independent tests (corresponding to the five kidney-related traits tested), resulting in a p-value threshold of 0.01 (0.05/5).

For the local genetic correlation analysis, LAVA splits the autosomes into pre-defined 2495 regions/loci for testing based on LD patterns in the UK Biobank, as recommended by the software developers. In our LAVA analyses, we accounted for sample overlap by specifying the sampling correlation matrix from the results of cross-trait LDSC. First, LAVA carries out a univariate test to identify which loci have genetic signal by testing for per-trait local heritability. Only loci with adequate signal upon applying a Bonferroni-corrected *p*-value threshold, *p* < 0.05/2459, in the univariate test for both traits proceed to the bivariate (genetic correlation) test. We applied a Bonferroni threshold at the univariate and bivariate stages based on the number of loci tested. For the univariate test, the *p*-value threshold was set to 2.0 × 10^−5^ (0.05 divided by 2495, the number of pre-defined loci), meaning a particular locus only proceeded to the bivariate genetic correlation test if both traits passed this significance threshold. For the bivariate (genetic correlation) test, the significance threshold was determined for each pairwise analysis based on the number of loci passing the univariate test for both traits. Specifically, for each pair of traits tested, the *p*-value threshold was set to 0.05 divided by the number of loci tested in the bivariate test.

### Identification of shared genomic loci

We then identified genetic loci jointly associated with PD and kidney traits through conjunctional false discovery rate (conjFDR) analysis using the pleioFDR software v1.0 (https://github.com/precimed/pleiofdr)^29^. LAVA and conjFDR address complementary questions. LAVA estimates the direction-dependent local genetic correlation within LD blocks, whereas conjFDR identifies discrete loci jointly associated with both traits independent of effect direction. As our aim was to identify specific shared loci, we used conjFDR as the primary locus-discovery method.

ConjFDR is a Bayesian method that estimates the posterior probability that a SNP is non-null for both phenotypes simultaneously, leveraging cross-trait enrichment to boost power while remaining agnostic to direction of effect. For each PD-kidney trait pair, conjFDR is defined as the maximum of the two conditional FDR values (i.e., conjFDR = max[condFDR₁|₂, condFDR₂|₁]), ensuring that a SNP is declared significant only if it shows evidence of association with both traits. GWAS summary statistics were converted to pleioFDR-compatible.mat format using the python_convert toolset, with SNPs matched by rsID to the 1000 Genomes Phase 3 European reference panel. Random pruning was performed across 500 iterations at an r^2^ threshold of 0.1 to approximate SNP independence. We tested 15 strata-matched PD-kidney trait pairs: PD × 5 kidney phenotypes, matching sex-combined with sex-combined and sex-stratified with sex-stratified strata. Significant shared loci were defined at conjFDR <0.05, consistent with established conventions^29^. The conjFDR framework inherently controls for multiple testing across all SNPs within each trait pair through its empirical Bayesian FDR estimation. Loci in the major histocompatibility complex (MHC) region (chr6:25–34 Mb, GRCh37) were reported but not followed up in downstream analyses due to extended long-range LD.

### Colocalization analysis

For each PD-kidney trait pair, to assess whether shared associations identified by conjFDR reflect a common causal variant between the two tested traits or distinct variants in LD, we performed Bayesian colocalization using coloc.abf, as implemented in the coloc R package (v5.2.3)^31^. coloc.abf derives colocalization posteriors directly from the marginal association statistics under the assumption of at most one causal variant per trait. We applied it at every non-MHC conjFDR locus across the seven PD-kidney trait pairs, extracting summary statistics within ±500 kb of the lead variant for both traits. Prior probabilities were set conservatively (*p*_1_ = *p*_2_ = 1 × 10⁻⁴, *p*_12_ = 5 × 10⁻⁶) to reduce false-positive colocalization; the sensitivity of the calls to this choice was assessed across a grid of prior values (Supplementary Data 20). The method evaluates five mutually exclusive hypotheses: no association (H0), association with PD only (H1), the kidney trait only (H2), both traits through distinct causal variants (H3), or a single shared causal variant (H4). We considered a locus to show strong evidence of a shared causal variant when PP.H4 > 0.80, loci with PP.H3 > 0.80 were classified as harboring distinct causal variants. Because the same region could be identified in more than one trait pair or stratum, we collapsed loci within 500 kb of each other into distinct genomic loci for reporting. For each colocalized locus, the variant with the highest posterior probability of being the shared causal variant (SNP.PP.H4) was designated the candidate shared variant and queried against GTEx v11 cis-eQTL data in available brain and kidney tissues and, for the kidney, the NephQTL2 glomerular and tubulointerstitial resource^32^; genes with a significant eGene-level association (FDR < 0.05) and a significant single-variant cis-association (Bonferroni *P* < 8.3 × 10⁻⁴) were annotated as candidate effector genes.

### Functional enrichment analysis

For each trait pair with at least one shared locus (conjFDR <0.05), shared loci were annotated to protein-coding genes and tested for functional over-representation using two complementary frameworks. First, input gene lists were tested for over-representation against all gene-set collections available in FUMA GENE2FUNC v2.1.6^33^. *P* values were corrected using the Benjamini-Hochberg procedure; gene sets with a false discovery rate (FDR) > 0.05 were considered significant. Second, to place the shared genes in the context of protein interactions, protein-protein interaction (PPI) networks were constructed in NetworkAnalyst 3.0^34^ using the IMEx Interactome^35^, and a minimum-connected module was extracted for each input gene set using the prize-collecting Steiner forest (PCSF) algorithm^36^. Functional enrichment of each module was evaluated against the KEGG, Reactome, and Gene Ontology collections by hypergeometric test with Benjamini-Hochberg correction (FDR < 0.05). Genes within the MHC region were removed prior to enrichment to prevent the extended LD and high gene density of this region from dominating the results.

### Reporting summary

Further information on research design is available in the Nature Portfolio Reporting Summary linked to this article.

## Supplementary information

Supplementary Information

Description of Additional Supplementary Files

Supplementary Data 1-21

Reporting Summary

Transparent Peer Review file

## Source data

Source Data

## Data Availability

This study analyzed genome-wide association study summary statistics and individual-level UK Biobank data; no new participant data were collected. Sex-stratified PD summary statistics (Blauwendraat et al.) are available at [https://personal.broadinstitute.org/ryank/Blauwendraat_Parkinsons_sexdiff_2021.zip]. Sex-combined estimated glomerular filtration rate summary statistics from the CKDGen Consortium and UK Biobank meta-analysis (Stanzick et al.) are available at [https://ckdgen.imbi.uni-freiburg.de/]. The sex-stratified estimated glomerular filtration rate summary statistics have been deposited in Zenodo https://doi.org/10.5281/zenodo.22150292. Genome-wide sex-specific summary statistics for hematuria are available at [https://my.locuszoom.org/gwas/496796/] (female-specific) and [https://my.locuszoom.org/gwas/494413/] (male-specific). Sex-combined summary statistics for hematuria are available [https://pheweb.org/UKB-SAIGE/pheno/593]. The sex-stratified and sex-combined urinary albumin-to-creatinine ratio, urinary potassium-to-creatinine ratio, urinary sodium-to-creatinine ratio summary statistics generated in this study from the UK Biobank have been deposited in Zenodo https://doi.org/10.5281/zenodo.22150292. UK Biobank individual-level data are available under restricted access because access is governed by the UK Biobank Access Management System; access can be obtained by submitting a research application [https://www.ukbiobank.ac.uk/enable-your-research/apply-for-access]. Expression quantitative trait locus data were obtained from GTEx v11 [https://gtexportal.org/home/downloads/adult-gtex] and NephQTL2 [https://nephqtl2.org]. LD reference data were obtained from the 1000 Genomes Project Phase 3 European reference panel [https://www.internationalgenome.org/data]. Gene sets were queried through FUMA v2.1.6 [https://fuma.ctglab.nl] and NetworkAnalyst 3.0 [https://www.networkanalyst.ca]. Source data are provided with this paper.

## Code availability

The code used for this work is available on GitHub: https://github.com/GaglianoTaliun-Lab/kidney-neurodegenhttps://doi.org/10.5281/zenodo.22150293.
