# Supplementary material for: Genome-wide cross-trait analysis to compare genetic architecture between Parkinson’s disease and kidney-related traits
Source: Nat Commun. 2026 Sep 18;17:10966. doi: 10.1038/s41467-026-77920-3 (PMC13683393; doi:10.1038/s41467-026-77920-3)
Supplement: Supplementary file 1 [file 41467_2026_77920_MOESM1_ESM.pdf]

Supplementary Information

Genome-wide cross-trait analysis to compare genetic architecture between Parkinson's disease and kidney-related traits

Supplementary Methods

1. Genome-wide association study summary statistics

We assembled sex-combined and sex-stratified genome-wide association study (GWAS) summary statistics for Parkinson's disease (PD) and five kidney-related traits, all derived from individuals of European genetic ancestry and aligned to genome build GRCh37/hg19 (Table 1).

Parkinson's disease. Genetic studies of late-onset neurodegenerative disease frequently augment clinically ascertained case–control GWAS with "proxy" cases—unaffected individuals who report an affected first-degree relative—to increase effective sample size. However, proxy-based GWAS of Alzheimer's disease have recently been shown to introduce systematic biases that distort downstream genetic-correlation and pleiotropy estimates[1], and the proportion and definition of proxy cases differ between PD studies, introducing an additional source of heterogeneity. To eliminate this bias, all analyses used PD summary statistics that exclude proxy cases. Sex-stratified PD GWAS (female, 7,947 cases / 20,330 controls; male, 13,020 cases / 19,336 controls) were obtained from Blauwendraat et al.[2]; controls were participants with neither a PD diagnosis nor a reported parental history of PD and an age of recruitment exceeding 60 years. Because both strata were generated under a uniform ascertainment scheme, we constructed proxy-free sex-combined statistics by fixed-effects, inverse-variance-weighted meta-analysis of the female- and male-specific datasets in METAL[3], combining per-variant effect-size estimates and standard errors. This yielded a sex-combined PD dataset (20,967 cases / 39,666 controls) free of the proxy-inclusion heterogeneity present in published sex-combined PD meta-analyses.

Kidney-related traits. We selected five phenotypes capturing complementary aspects of renal function and urinary physiology (Table 1). Sex-combined estimated glomerular filtration rate (eGFR) statistics were drawn from the CKDGen Consortium and UK Biobank meta-analysis of Stanzick et al.[4] (N = 1,201,930), with eGFR estimated from serum creatinine using the CKD-EPI 2009 equation[5]. Sex-stratified eGFR, urinary albumin-to-creatinine ratio (uACR), urinary potassium-to-creatinine ratio (uK/Cr) and urinary sodium-to-creatinine ratio (uNa/Cr) GWAS were performed in UK Biobank White British participants, adjusting for age and genetic principal components (and additionally for sex in sex-combined analyses). Hematuria was defined from ICD diagnosis codes as described previously[6]. To remove variation attributable to urine concentration, urinary electrolytes were normalized to urinary creatinine, yielding dilution-corrected ratios; uACR is intrinsically dilution-corrected, with left-censored albumin values (<6.7 mg/L) set to 6.7 mg/L. Dilution correction of the urinary biomarkers was implemented in response to peer review.

2. Estimation of SNP-based heritability

We estimated the SNP-based heritability of each trait using univariate LD Score regression (LDSC)[7]. Under a polygenic model, LDSC exploits the expected relationship between a variant's

association test statistic and its LD score ℓ_j (the sum of squared correlations between variant j and all neighbouring variants): $E[\chi^2_j] = 1 + Na + (Nh^2/M)\ \ell_j$, where N is the GWAS sample size, M the number of variants, h^2 the SNP-based heritability and a term capturing confounding from population stratification or cryptic relatedness. Regressing the observed χ^2 statistics on LD scores therefore yields a slope proportional to h^2 and an intercept that departs from unity only in the presence of confounding. LD scores were computed from the 1000 Genomes Phase 3 European reference panel; analyses were restricted to HapMap3 variants and excluded the MHC region. Heritability is reported on the observed scale together with the genomic inflation factor (λ_{GC}), mean χ^2 and LDSC intercept. Intercepts close to one indicate that test-statistic inflation reflects polygenic signal rather than confounding; intercepts marginally below one for PD reflect the conservative behaviour of LDSC at the sample sizes analysed.

3. Genome-wide genetic correlation

Pairwise genome-wide genetic correlations were estimated using bivariate (cross-trait) LD Score regression[7,8]. Extending the univariate model, the expected product of two traits' association z-scores at variant j is $E[z_{1j}\ z_{2j}] = (\sqrt{(N_1N_2)}\ \rho_g/M)\ \ell_j + (N_s\ \rho/\sqrt{(N_1N_2)})$, where ρ_g is the genome-wide genetic covariance, N_1 and N_2 the per-trait sample sizes, N_s the number of overlapping individuals and ρ their phenotypic correlation. The regression slope provides an estimate of ρ_g, from which the genetic correlation is obtained as $r_g = \rho_g/\sqrt{(h_1^2\ h_2^2)}$ and is bounded to $[-1, 1]$. Importantly, the regression intercept absorbs the contribution of sample overlap and phenotypic correlation, so unbiased genetic correlations can be estimated even when the degree of participant overlap between the PD and kidney GWAS is unknown. We tested 15 strata-matched PD-kidney trait pairs (sex-combined with sex-combined, and each sex-stratified pair within stratum). Each genetic correlation was tested against the null of $r_g = 0$ using a two-sided Z-test, and significance was assessed against a Bonferroni-corrected threshold of $P < 3.3 \times 10^{-3}$ (0.05/15).

4. Local genetic correlation

Genome-wide genetic correlation can obscure genetic sharing that is heterogeneous or confined to specific regions. We therefore estimated local genetic correlations using Local Analysis of [co]Variant Association (LAVA)[9]. LAVA partitions the autosomes into 2,495 approximately LD-independent blocks defined from a population reference. Within each block, the local genetic signal for a trait is modelled from the joint variant effects; because neighbouring variants are correlated, the local LD matrix is decomposed by principal-component analysis and only the leading components jointly explaining at least 99% of the genetic variance are retained, mitigating collinearity and reducing dimensionality. LAVA proceeds in two stages. First, a univariate test estimates the local SNP-based heritability of each trait and evaluates whether it differs from zero; a block proceeds only if both traits exhibit significant local heritability at a Bonferroni-corrected threshold of $P < 2.0 \times 10^{-5}$ (0.05/2,495). Second, for qualifying blocks, the local genetic correlation

ρ is computed as the local genetic covariance divided by the square root of the product of the two local heritabilities and is bounded to [−1, 1]. Sample overlap between the PD and kidney GWAS was accounted for by supplying the sampling-correlation matrix obtained from the intercepts of cross-trait LDSC. The significance of each bivariate test was assessed against a Bonferroni threshold defined per pairwise analysis as 0.05 divided by the number of blocks passing the univariate filter for both traits.

5. Identification of shared genomic loci

To localize specific variants jointly associated with PD and each kidney trait, we applied the conjunctional false discovery rate (conjFDR) framework implemented in pleioFDR[10,11]. The approach builds on the conditional FDR (condFDR), which leverages cross-trait enrichment: if variants associated with a conditioning trait are enriched for associations with a primary trait, conditioning on the secondary trait re-ranks the primary-trait evidence and increases discovery power. The condFDR of a variant for the primary trait given the conditioning trait, condFDR($trait_1 \mid trait_2$), is the empirical posterior probability that the variant is null for trait 1, given that its trait-1 and trait-2 P-values are at least as extreme as those observed; it is estimated from the two-dimensional empirical conditional cumulative distribution of P-values across all variants. To require evidence of association with both phenotypes and to remove dependence on which trait is treated as primary, the conjunctional FDR is defined as the maximum of the two reciprocal conditional values, conjFDR = max[condFDR($trait_1 \mid trait_2$), condFDR($trait_2 \mid trait_1$)]; this is a conservative estimate of the FDR for the composite null that a variant is associated with at most one of the two traits. Because LD inflates the apparent number of independent associations and biases FDR estimation, variants were randomly pruned over 500 iterations at an LD threshold of $r^2 < 0.1$ using the 1000 Genomes Phase 3 European reference panel, and conditional FDR values were averaged across iterations. The method is agnostic to direction of effect. Summary statistics were converted to the required format with the python_convert toolset, matching variants to the reference panel by rsID; strand-ambiguous variants for which strand could not be resolved were assigned missing z-scores by design and excluded from FDR estimation. Shared loci were declared at conjFDR < 0.05. Variants in the extended MHC region (chr6:25-34 Mb) were reported but excluded from downstream analyses owing to its extended linkage disequilibrium and high gene density.

6. Bayesian colocalization

For each non-MHC conjFDR locus, we used Bayesian colocalization to distinguish a single shared causal variant from distinct variants in linkage disequilibrium between PD and the kidney trait. Summary statistics for both traits were extracted within ±500 kb of the locus lead variant. Colocalization was performed with coloc.abf, as implemented in the coloc package (v5.2.3)[12]. coloc.abf derives colocalization posteriors directly from the marginal association statistics — each

variant's approximate Bayes factor, from its association z-score and a prior on the effect-size variance — under the assumption of at most one causal variant per trait. For a region, coloc evaluates five mutually exclusive hypotheses — no association (H0), association with trait 1 only (H1), trait 2 only (H2), both traits via distinct causal variants (H3), or both via one shared causal variant (H4) — combining each variant's Bayes factor with prior probabilities that a variant is causal for trait 1 (p1), trait 2 (p2) or both (p12). Prior probabilities were set conservatively ($p1 = p2 = 1\times10^{-4}$, $p12 = 5\times10^{-6}$, half the coloc default) to reduce spurious colocalization. A locus was considered to show strong evidence for a shared causal variant when PP.H4 > 0.80, and we additionally report loci meeting a composite criterion ($PP.H3 + PP.H4 \geq 0.8$ and $PP.H4/PP.H3 \geq 5$); loci with PP.H3 > 0.80 were classified as harbouring distinct causal variants. Because the same region could be identified in more than one trait pair or stratum, loci within 500 kb were collapsed into distinct genomic loci, yielding 84 distinct non-MHC loci. For each colocalized locus, the variant with the highest posterior probability of being the shared causal variant (SNP.PP.H4) was taken as the candidate shared causal variant.

7. cis-eQTL annotation of candidate shared variants

To move from positional to regulatory gene assignment, each top candidate shared causal variant was queried against cis-expression quantitative trait locus (cis-eQTL) data in the Genotype-Tissue Expression project (GTEx v11), focusing on PD-relevant brain tissues and kidney cortex. For each variant-gene-tissue association, we recorded the normalized effect size (NES) and association P-value. Because GTEx kidney cortex is sampled shallowly, kidney annotation was strengthened using NephQTL2[13], a compartment-resolved kidney cis-eQTL resource from the Nephrotic Syndrome Study Network (NEPTUNE) built from 240 glomerular and 311 tubulointerstitial micro-dissected human kidney biopsy samples. NephQTL2 identifies eGenes by gene-level Bayesian FDR and fine-maps each signal with DAP-G under an integrative prior incorporating kidney single-nucleus open-chromatin annotations and transcription start site distance, yielding higher-resolution kidney eGenes than bulk kidney cortex. Each variant was queried in both the glomerular and tubulointerstitial compartments, and we recorded its cis-eQTL effect and the corresponding eGene-level significance. Variants displaying significant eQTL effects in either resource were annotated to the corresponding target gene, refining assignments based solely on physical proximity.

8. Functional enrichment analysis

Locus definition and gene mapping. For each trait pair, independent genomic loci were defined from the conjFDR results by LD-clumping. Independent significant variants were identified at $r^2 < 0.6$, lead variants at $r^2 < 0.1$, and lead variants separated by less than 250 kb were merged into a single locus. Protein-coding genes (GENCODE v37) located within each locus and a ±10 kb flanking window were mapped as candidate genes.

Gene-set over-representation. Mapped protein-coding genes for each trait pair were tested for over-representation against the gene-set collections in FUMA GENE2FUNC[14] using a hypergeometric test; enrichment P-values were corrected by the Benjamini-Hochberg procedure, and gene sets with FDR < 0.05 were considered significant.

Protein-protein interaction network enrichment. To interpret the mapped genes in the context of physical interactions, protein-protein interaction (PPI) networks were constructed in NetworkAnalyst[15] using the IMEx interactome[16]. For each trait pair's gene set, a minimal connected subnetwork was extracted using the prize-collecting Steiner forest (PCSF) algorithm[17], which identifies a low-cost connected module by maximizing the total "prize" of included seed genes while penalizing the number and cost of edges, thereby linking input genes through a parsimonious set of intermediate (connector) proteins. Functional enrichment of the resulting module genes was evaluated against the KEGG, Reactome and Gene Ontology (Biological Process and Molecular Function) collections by hypergeometric test with Benjamini-Hochberg correction (FDR < 0.05). Because PCSF modules include both seed genes and algorithmically inferred connector genes, enriched pathways reflect the broader network neighbourhood of the shared loci rather than the input genes alone. Variants and genes in the MHC region were removed prior to all enrichment analyses to prevent the region's extended linkage disequilibrium and high gene density from dominating the results.

Supplementary Note

1. Sensitivity analysis using cystatin C-based eGFR

Serum creatinine, from which eGFR is derived, depends in part on skeletal muscle mass, so a genetic relationship between PD and creatinine-based eGFR could in principle reflect muscle-mass biology. To evaluate this, we repeated the PD-eGFR analysis using cystatin C-based eGFR (eGFRcys), a glomerular filtration marker that is largely independent of muscle mass. Sex-combined eGFRcys summary statistics (n = 460,826; individuals of European ancestry) were obtained from Stanzick et al. (2021)[4]. We applied the same pipeline used for the primary analysis: conjunctional FDR (conjFDR < 0.05) to identify loci jointly associated with PD and eGFRcys, followed by Bayesian colocalization (coloc.abf; priors $p1 = p2 = 1\times10^{-4}$, $p12 = 5\times10^{-6}$) at each non-MHC locus to distinguish shared (H4) from distinct (H3) causal variants, with all thresholds as in the main analysis.

In this analysis the *BIN3* locus colocalized with eGFRcys (PP.H4 = 0.87; candidate shared variant rs13264187), recovering its colocalization with creatinine-based eGFR, while *SCARB2/FAM47E-STBD1* remained distinct (PP.H3 = 0.9999). Because cystatin C is largely independent of muscle mass and eGFRcys is derived predominantly (~95%) from a single cohort (UK Biobank, n =

436,765; with ~5% from the CKDGen European meta-analysis), this recovery indicates that the *BIN3* shared signal is not an artefact of the muscle-mass component of creatinine or of cross-cohort meta-analysis.

This eGFRcys analysis is a sensitivity analysis and is not included in the main-text colocalization counts (128 locus–trait-pair tests across 70 distinct non-MHC loci in the seven primary trait pairs); including it, Supplementary Data 12 lists 175 tests across 84 distinct loci, of which 47 tests and 14 additional loci derive from the PD-eGFRcys pair.

2. Prior sensitivity of colocalization

Because coloc.abf posteriors depend on the choice of prior, we assessed the sensitivity of every colocalized locus to the prior probabilities. Colocalization was repeated across a grid of p12 values (1×10^{-6}, 5×10^{-6} [primary], 1×10^{-5} [the coloc default] and 5×10^{-5}) and at a less-conservative setting ($p1 = p2 = 1\times10^{-3}$, $p12 = 1\times10^{-4}$). The shared-versus-distinct classification was stable across the primary and less-conservative priors, with the evidence favouring a shared over a distinct causal variant at every shared locus and prior; only at the most conservative setting ($p12 = 1\times10^{-6}$) did the most borderline loci become inconclusive rather than shared, and no shared locus was reclassified as distinct. Full results are reported in Supplementary Data 20.

3. Diagnostic for meta-analysis fine-mapping (SLALOM)

Because both discovery GWAS are themselves meta-analyses, and fine-mapping of meta-analysis summary statistics can be miscalibrated[18], we screened every reported colocalized locus for the summary-statistic/LD inconsistency that flags this problem, using SLALOM[18], the diagnostic introduced for this purpose. SLALOM identifies variants whose association statistics are inconsistent with the locus lead given local LD, using a simplified DENTIST test (DENTIST-S; Chen et al., 2021); a variant is flagged as an outlier when it is in LD with the lead ($r^2 > 0.6$) and its DENTIST-S $P < 1\times10^{-4}$. Following Kanai et al., the LD reference is used to diagnose summary-statistic/LD inconsistency. We built a large in-sample UK Biobank LD reference from the genotyping array (N = 337,462 unrelated individuals of white-British ancestry, defined from the sample-QC file), and ran DENTIST-S for both traits at every reported colocalized locus and at the *SCARB2/FAM47E-STBD1* distinct-variant control. Across all reported colocalized loci (18 locus-trait tests), SLALOM identified no DENTIST-S outlier variants; a single near-threshold outlier arose only at the *SCARB2/FAM47E-STBD1* distinct-variant control, at a variant with posterior inclusion probability ≈ 0 that is neither the lead variant nor in the 95% credible set. Because the in-sample panel is array-based, it is best powered where the lead variant has many LD partners (e.g. *BIN3*, 26 variants) and thinner where few variants exceed $r^2 > 0.6$ with the lead (e.g. *RAB19*, *TNK2*); the diagnostic is therefore a screen for a specific inconsistency rather than a certification of posterior calibration. Full results are reported in Supplementary Data 21.

4. conjFDR threshold sensitivity

To assess whether the PD-eGFR signal — by far the largest, reflecting the power of the eGFR GWAS — is inflated by the permissiveness of the conjFDR threshold, we recomputed the number of independent non-MHC PD-eGFR loci at progressively stricter thresholds. The count falls from 58 at conjFDR < 0.05 to 16 at < 0.01 to 8 at < 0.001, and the core loci (*FAM47E-STBD1/SCARB2, CTSB*, and *TMEM163*) persist at the strictest threshold (Supplementary Data 19). Because the conjunctional FDR is the maximum of the two conditional FDRs and therefore a conservative estimate of the true FDR, additional loci recovered at higher power reflect additional true discoveries rather than false positives; the guard against false shared-signal claims is the colocalization step, at which distinct variants outnumber shared, not the conjFDR count.

Supplementary Figure

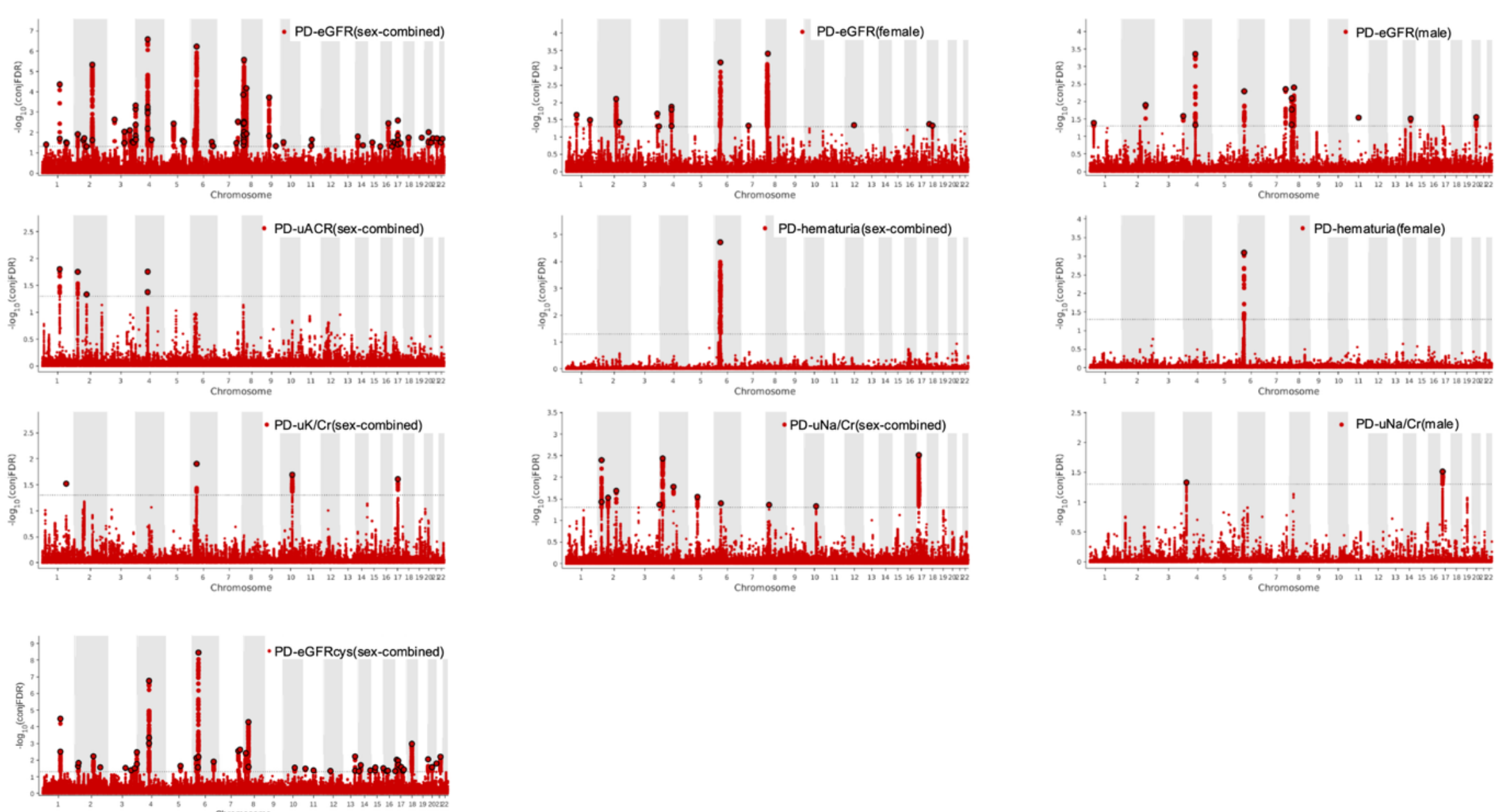

Supplementary Figure 1. Conjunctional FDR Manhattan plots for Parkinson's disease paired with kidney-related traits. a–j, conjunctional false discovery rate (conjFDR) Manhattan plots for the 10 of the 16 PD–kidney trait pairs tested (the 15 primary trait pairs plus the PD–eGFRcys sensitivity analysis) that yielded at least one locus at conjFDR < 0.05. a, PD–eGFR (sex-combined); b, PD–eGFR (female-only); c, PD–eGFR (male-only); d, PD–uACR (sex-combined); e, PD–hematuria (sex-combined); f, PD–hematuria (female-only); g, PD–uK/Cr (sex-combined); h, PD–uNa/Cr (sex-combined); i, PD–uNa/Cr (male-only); j, PD–eGFRcys (sex-combined). In every panel the x-axis gives chromosomal position (GRCh37/hg19), with alternating grey and white vertical shading distinguishing consecutive chromosomes, and the y-axis gives −log10(conjFDR) for each SNP; y-axis scales differ between panels. Each red point is one SNP

and the colour carries no quantitative information, so no colour scale applies. The horizontal dashed line marks the conjFDR = 0.05 significance threshold (−log10 = 1.30), and black-outlined points mark the lead SNP of each LD-independent locus reaching conjFDR < 0.05. Points show single-variant statistics rather than summarised data, so no bar graphs, error bars, measures of centre or dispersion, scale bars or molecular-weight markers apply, and no data are presented as bar charts. conjFDR values were computed with pleioFDR from the exact two-sided association P values of the two source genome-wide association studies, as conjFDR = max[condFDR(trait 1 | trait 2), condFDR(trait 2 | trait 1)], after random linkage-disequilibrium pruning (r2 < 0.1, 1000 Genomes Phase 3 European reference panel) averaged over 500 iterations. conjFDR is itself the adjustment for multiple testing across the genome and no further correction was applied; because it is a false-discovery-rate procedure rather than a null-hypothesis significance test, no test statistic, degrees of freedom or confidence interval is defined for it. Exact conjFDR values, positions and lead SNPs for every locus reaching conjFDR < 0.05 are given in Supplementary Data 3–11 (panels a–i) and Supplementary Data 18 (panel j). Sample sizes are those of the source genome-wide association studies; no additional participants were analysed for this figure, the analysis is deterministic and involves no experimental replication, and the unit of study is the individual participant. Parkinson's disease: n = 60,633 individuals (20,967 cases, 39,666 controls) sex-combined, n = 28,277 (7,947 cases, 20,330 controls) female-only, n = 32,356 (13,020 cases, 19,336 controls) male-only. Kidney traits: eGFR n = 1,201,930 individuals (sex-combined), n = 209,516 (female-only), n = 178,711 (male-only); uACR n = 122,972 individuals (sex-combined); hematuria n = 396,345 individuals (16,409 cases, 379,936 controls) sex-combined, n = 220,094 (7,061 cases, 213,033 controls) female-only; uK/Cr n = 394,601 individuals (sex-combined); uNa/Cr n = 394,639 (sex-combined), n = 182,099 (male-only) individuals; eGFRcys n = 460,826 individuals (sex-combined). Sample sizes and data sources for every dataset are listed in Table 1 of the main article. eGFR, creatinine-based estimated glomerular filtration rate; eGFRcys, cystatin C-based estimated glomerular filtration rate; LD, linkage disequilibrium; PD, Parkinson's disease; SNP, single-nucleotide polymorphism; uACR, urinary albumin-to-creatinine ratio; uK/Cr, urinary potassium-to-creatinine ratio; uNa/Cr, urinary sodium-to-creatinine ratio.

Supplementary References

1 Wu, Y. *et al.* Pervasive biases in proxy genome-wide association studies based on parental history of Alzheimer's disease. *Nat Genet* **56**, 2696-2703 (2024). https://doi.org:10.1038/s41588-024-01963-9

2 Blauwendraat, C. *et al.* Investigation of Autosomal Genetic Sex Differences in Parkinson's Disease. *Ann Neurol* **90**, 35-42 (2021). https://doi.org:10.1002/ana.26090

3 Willer, C. J., Li, Y. & Abecasis, G. R. METAL: fast and efficient meta-analysis of genomewide association scans. *Bioinformatics* **26**, 2190-2191 (2010). https://doi.org:10.1093/bioinformatics/btq340

4 Stanzick, K. J. *et al.* Discovery and prioritization of variants and genes for kidney function in >1.2 million individuals. *Nat Commun* **12**, 4350 (2021). https://doi.org:10.1038/s41467-021-24491-0

5 Levey, A. S. *et al.* A new equation to estimate glomerular filtration rate. *Ann Intern Med* **150**, 604-612 (2009). https://doi.org:10.7326/0003-4819-150-9-200905050-00006

6 Gagliano Taliun, S. A., Dinsmore, I. R., Mirshahi, T., Chang, A. R., Paterson, A. D. & Barua, M. GWAS for the composite traits of hematuria and albuminuria. *Sci Rep* **13**, 18084 (2023). https://doi.org:10.1038/s41598-023-45102-6

7 Bulik-Sullivan, B. K. *et al.* LD Score regression distinguishes confounding from polygenicity in genome-wide association studies. *Nat Genet* **47**, 291-295 (2015). https://doi.org:10.1038/ng.3211

8 Bulik-Sullivan, B. *et al.* An atlas of genetic correlations across human diseases and traits. *Nat Genet* **47**, 1236-1241 (2015). https://doi.org:10.1038/ng.3406

9 Werme, J., van der Sluis, S., Posthuma, D. & de Leeuw, C. A. An integrated framework for local genetic correlation analysis. *Nat Genet* **54**, 274-282 (2022). https://doi.org:10.1038/s41588-022-01017-y

10 Smeland, O. B. *et al.* Discovery of shared genomic loci using the conditional false discovery rate approach. *Hum Genet* **139**, 85-94 (2020). https://doi.org:10.1007/s00439-019-02060-2

11 Andreassen, O. A. *et al.* Improved detection of common variants associated with schizophrenia and bipolar disorder using pleiotropy-informed conditional false discovery rate. *PLoS Genet* **9**, e1003455 (2013). https://doi.org:10.1371/journal.pgen.1003455

12 Wallace, C. A more accurate method for colocalisation analysis allowing for multiple causal variants. *PLoS Genet* **17**, e1009440 (2021). https://doi.org:10.1371/journal.pgen.1009440

13 Han, S. K. *et al.* Mapping genomic regulation of kidney disease and traits through high-resolution and interpretable eQTLs. *Nat Commun* **14**, 2229 (2023). https://doi.org:10.1038/s41467-023-37691-7

14 Watanabe, K., Taskesen, E., van Bochoven, A. & Posthuma, D. Functional mapping and annotation of genetic associations with FUMA. *Nat Commun* **8**, 1826 (2017). https://doi.org:10.1038/s41467-017-01261-5

15 Zhou, G., Soufan, O., Ewald, J., Hancock, R. E. W., Basu, N. & Xia, J. NetworkAnalyst 3.0: a visual analytics platform for comprehensive gene expression profiling and meta-analysis. *Nucleic Acids Res* **47**, W234-w241 (2019). https://doi.org:10.1093/nar/gkz240

16 Orchard, S. *et al.* Protein interaction data curation: the International Molecular Exchange (IMEx) consortium. *Nat Methods* **9**, 345-350 (2012). https://doi.org:10.1038/nmeth.1931

17 Akhmedov, M. *et al.* PCSF: An R-package for network-based interpretation of high-throughput data. *PLoS Comput Biol* **13**, e1005694 (2017). https://doi.org:10.1371/journal.pcbi.1005694

18 Kanai, M., Elzur, R., Zhou, W., Daly, M. J. & Finucane, H. K. Meta-analysis fine-mapping is often miscalibrated at single-variant resolution. *Cell Genom* **2** (2022). https://doi.org:10.1016/j.xgen.2022.100210
