# Supplementary material for: Genome-wide cross-trait analysis to compare genetic architecture between Parkinson’s disease and kidney-related traits
Source: Nat Commun. 2026 Sep 18;17:10966. doi: 10.1038/s41467-026-77920-3 (PMC13683393; doi:10.1038/s41467-026-77920-3)
Supplement: Supplementary file 2 [file 41467_2026_77920_MOESM2_ESM.pdf]

Description of Additional Supplementary Files

Supplementary Data 1. **SNP-based heritability estimates from LD Score regression**. SNP-based heritability (h^2_obs) was estimated using LD Score regression with the 1000 Genomes Phase 3 European LD reference panel. Lambda_GC: genomic inflation factor. Mean_chi2: mean chi-squared statistic. Intercept: LDSC intercept (values close to 1 indicate minimal confounding; values <1 for PD reflect conservative correction). SE: standard error.

Supplementary Data 2. **Genome-wide genetic correlations between Parkinson's disease and kidney-related traits**. Genetic correlations (rg) were estimated using bivariate LD Score regression across 15 PD–kidney trait pairs. Bonferroni-corrected significance threshold: $P < 0.0033$ (0.05/15). SE: standard error. Z: Z-score. P: two-sided P-value.

Supplementary Data 3. **conjunctional FDR results for SNPs reaching conjFDR < 0.05 for PD (sex-combined) × eGFR (sex-combined)**. conjunctional FDR (conjFDR) results for all SNPs reaching conjFDR < 0.05, from pleioFDR (Smeland et al., 2020; Andreassen et al., 2013) using a 1000 Genomes Phase 3 European LD reference and 500 random-pruning iterations at $r2 < 0.1$; conjFDR is the maximum of the two conditional FDRs. Columns: locusnum, conjFDR locus number (SNPs grouped into physically contiguous loci); rsid, dbSNP reference SNP ID; chrnum and chrpos, chromosome and base-pair position (GRCh37); zscore_* columns, GWAS z-scores for Parkinson's disease and creatinine-based eGFR; conjfdr_* , conjunctional FDR; prune_*, random-pruning indicator (1 marks the SNP retained as an LD-independent representative after random pruning, 0 otherwise); min_conjfdr, the minimum conjFDR value (identical to the conjfdr column for all SNPs reported here); pval_* columns, two-sided GWAS association p-values for the two traits. Column names carry the specific stratum/trait labels of this pair. Where present, MHC loci (chr6:25-34 Mb, GRCh37) are reported here but excluded from all downstream (colocalization and enrichment) analyses.

Supplementary Data 4. **conjunctional FDR results for SNPs reaching conjFDR < 0.05 for PD (sex-combined) × uACR (sex-combined)**. conjunctional FDR (conjFDR) results for all SNPs reaching conjFDR < 0.05, from pleioFDR (Smeland et al., 2020; Andreassen et al., 2013) using a 1000 Genomes Phase 3 European LD reference and 500 random-pruning iterations at $r2 < 0.1$; conjFDR is the maximum of the two conditional FDRs. Columns: locusnum, conjFDR locus number (SNPs grouped into physically contiguous loci); rsid, dbSNP reference SNP ID; chrnum and chrpos, chromosome and base-pair position (GRCh37); zscore_* columns, GWAS z-scores for Parkinson's disease and urinary albumin-to-creatinine ratio (uACR); conjfdr_* , conjunctional FDR; prune_*, random-pruning indicator (1 marks the SNP retained as an LD-independent representative after random pruning, 0 otherwise); min_conjfdr, the minimum conjFDR value (identical to the conjfdr column for all SNPs reported here); pval_* columns, two-sided GWAS association p-values for the two traits. Column names carry the specific stratum/trait labels of this pair. Where present, MHC loci (chr6:25-34 Mb, GRCh37) are

reported here but excluded from all downstream (colocalization and enrichment) analyses.

Supplementary Data 5. **conjunctional FDR results for SNPs reaching conjFDR < 0.05 for PD (sex-combined) × hematuria (sex-combined)**. conjunctional FDR (conjFDR) results for all SNPs reaching conjFDR < 0.05, from pleioFDR (Smeland et al., 2020; Andreassen et al., 2013) using a 1000 Genomes Phase 3 European LD reference and 500 random-pruning iterations at r2 < 0.1; conjFDR is the maximum of the two conditional FDRs. Columns: locusnum, conjFDR locus number (SNPs grouped into physically contiguous loci); rsid, dbSNP reference SNP ID; chrnum and chrpos, chromosome and base-pair position (GRCh37); zscore_* columns, GWAS z-scores for Parkinson's disease and hematuria; conjfdr_* , conjunctional FDR; prune_*, random-pruning indicator (1 marks the SNP retained as an LD-independent representative after random pruning, 0 otherwise); min_conjfdr, the minimum conjFDR value (identical to the conjfdr column for all SNPs reported here); pval_* columns, two-sided GWAS association p-values for the two traits. Column names carry the specific stratum/trait labels of this pair. Where present, MHC loci (chr6:25-34 Mb, GRCh37) are reported here but excluded from all downstream (colocalization and enrichment) analyses.

Supplementary Data 6. **conjunctional FDR results for SNPs reaching conjFDR < 0.05 for PD (sex-combined) × uKCr (sex-combined)**. conjunctional FDR (conjFDR) results for all SNPs reaching conjFDR < 0.05, from pleioFDR (Smeland et al., 2020; Andreassen et al., 2013) using a 1000 Genomes Phase 3 European LD reference and 500 random-pruning iterations at r2 < 0.1; conjFDR is the maximum of the two conditional FDRs. Columns: locusnum, conjFDR locus number (SNPs grouped into physically contiguous loci); rsid, dbSNP reference SNP ID; chrnum and chrpos, chromosome and base-pair position (GRCh37); zscore_* columns, GWAS z-scores for Parkinson's disease and urinary potassium-to-creatinine ratio (uK/Cr); conjfdr_* , conjunctional FDR; prune_*, random-pruning indicator (1 marks the SNP retained as an LD-independent representative after random pruning, 0 otherwise); min_conjfdr, the minimum conjFDR value (identical to the conjfdr column for all SNPs reported here); pval_* columns, two-sided GWAS association p-values for the two traits. Column names carry the specific stratum/trait labels of this pair. Where present, MHC loci (chr6:25-34 Mb, GRCh37) are reported here but excluded from all downstream (colocalization and enrichment) analyses.

Supplementary Data 7. **conjunctional FDR results for SNPs reaching conjFDR < 0.05 for PD (sex-combined) × uNaCr (sex-combined)**. conjunctional FDR (conjFDR) results for all SNPs reaching conjFDR < 0.05, from pleioFDR (Smeland et al., 2020; Andreassen et al., 2013) using a 1000 Genomes Phase 3 European LD reference and 500 random-pruning iterations at r2 < 0.1; conjFDR is the maximum of the two conditional FDRs. Columns: locusnum, conjFDR locus number (SNPs grouped into physically contiguous loci); rsid, dbSNP reference SNP ID; chrnum and chrpos, chromosome and base-pair position (GRCh37); zscore_* columns, GWAS z-scores for Parkinson's disease and urinary sodium-to-creatinine ratio (uNa/Cr); conjfdr_* , conjunctional FDR; prune_*, random-pruning indicator (1 marks the SNP retained as an LD-independent

representative after random pruning, 0 otherwise); min_conjfdr, the minimum conjFDR value (identical to the conjfdr column for all SNPs reported here); pval_* columns, two-sided GWAS association p-values for the two traits. Column names carry the specific stratum/trait labels of this pair. Where present, MHC loci (chr6:25-34 Mb, GRCh37) are reported here but excluded from all downstream (colocalization and enrichment) analyses.

Supplementary Data 8. conjunctional FDR results for SNPs reaching conjFDR < 0.05 for PD (female) × eGFR (female). conjunctional FDR (conjFDR) results for all SNPs reaching conjFDR < 0.05, from pleioFDR (Smeland et al., 2020; Andreassen et al., 2013) using a 1000 Genomes Phase 3 European LD reference and 500 random-pruning iterations at r2 < 0.1; conjFDR is the maximum of the two conditional FDRs. Columns: locusnum, conjFDR locus number (SNPs grouped into physically contiguous loci); rsid, dbSNP reference SNP ID; chrnum and chrpos, chromosome and base-pair position (GRCh37); zscore_* columns, GWAS z-scores for Parkinson's disease and creatinine-based eGFR; conjfdr_* , conjunctional FDR; prune_*, random-pruning indicator (1 marks the SNP retained as an LD-independent representative after random pruning, 0 otherwise); min_conjfdr, the minimum conjFDR value (identical to the conjfdr column for all SNPs reported here); pval_* columns, two-sided GWAS association p-values for the two traits. Column names carry the specific stratum/trait labels of this pair. Where present, MHC loci (chr6:25-34 Mb, GRCh37) are reported here but excluded from all downstream (colocalization and enrichment) analyses.

Supplementary Data 9. **conjunctional FDR results for SNPs reaching conjFDR < 0.05 for PD (female) × hematuria (female)**. conjunctional FDR (conjFDR) results for all SNPs reaching conjFDR < 0.05, from pleioFDR (Smeland et al., 2020; Andreassen et al., 2013) using a 1000 Genomes Phase 3 European LD reference and 500 random-pruning iterations at r2 < 0.1; conjFDR is the maximum of the two conditional FDRs. Columns: locusnum, conjFDR locus number (SNPs grouped into physically contiguous loci); rsid, dbSNP reference SNP ID; chrnum and chrpos, chromosome and base-pair position (GRCh37); zscore_* columns, GWAS z-scores for Parkinson's disease and hematuria; conjfdr_* , conjunctional FDR; prune_*, random-pruning indicator (1 marks the SNP retained as an LD-independent representative after random pruning, 0 otherwise); min_conjfdr, the minimum conjFDR value (identical to the conjfdr column for all SNPs reported here); pval_* columns, two-sided GWAS association p-values for the two traits. Column names carry the specific stratum/trait labels of this pair. Where present, MHC loci (chr6:25-34 Mb, GRCh37) are reported here but excluded from all downstream (colocalization and enrichment) analyses.

Supplementary Data 10. **conjunctional FDR results for SNPs reaching conjFDR < 0.05 for PD (male) × eGFR (male)**. conjunctional FDR (conjFDR) results for all SNPs reaching conjFDR < 0.05, from pleioFDR (Smeland et al., 2020; Andreassen et al., 2013) using a 1000 Genomes Phase 3 European LD reference and 500 random-pruning iterations at r2 < 0.1; conjFDR is the maximum of the two conditional FDRs. Columns: locusnum, conjFDR locus number (SNPs grouped into physically contiguous loci); rsid, dbSNP reference SNP ID; chrnum and chrpos, chromosome and base-pair position

(GRCh37); zscore_* columns, GWAS z-scores for Parkinson's disease and creatinine-based eGFR; conjfdr_* , conjunctional FDR; prune_*, random-pruning indicator (1 marks the SNP retained as an LD-independent representative after random pruning, 0 otherwise); min_conjfdr, the minimum conjFDR value (identical to the conjfdr column for all SNPs reported here); pval_* columns, two-sided GWAS association p-values for the two traits. Column names carry the specific stratum/trait labels of this pair. Where present, MHC loci (chr6:25-34 Mb, GRCh37) are reported here but excluded from all downstream (colocalization and enrichment) analyses.

Supplementary Data 11. **conjunctional FDR results for SNPs reaching conjFDR < 0.05 for PD (male) × uNaCr (male)**. conjunctional FDR (conjFDR) results for all SNPs reaching conjFDR < 0.05, from pleioFDR (Smeland et al., 2020; Andreassen et al., 2013) using a 1000 Genomes Phase 3 European LD reference and 500 random-pruning iterations at r2 < 0.1; conjFDR is the maximum of the two conditional FDRs. Columns: locusnum, conjFDR locus number (SNPs grouped into physically contiguous loci); rsid, dbSNP reference SNP ID; chrnum and chrpos, chromosome and base-pair position (GRCh37); zscore_* columns, GWAS z-scores for Parkinson's disease and urinary sodium-to-creatinine ratio (uNa/Cr); conjfdr_* , conjunctional FDR; prune_*, random-pruning indicator (1 marks the SNP retained as an LD-independent representative after random pruning, 0 otherwise); min_conjfdr, the minimum conjFDR value (identical to the conjfdr column for all SNPs reported here); pval_* columns, two-sided GWAS association p-values for the two traits. Column names carry the specific stratum/trait labels of this pair. Where present, MHC loci (chr6:25-34 Mb, GRCh37) are reported here but excluded from all downstream (colocalization and enrichment) analyses.

Supplementary Data 12. **Bayesian colocalization (coloc.abf) of conjFDR-identified shared loci between Parkinson's disease and kidney-related traits**. Bayesian colocalization was performed with coloc.abf (Giambartolomei et al., 2014; implemented in the coloc R package, Wallace 2021) at every non-MHC conjFDR locus across eight PD-kidney trait pairs (175 locus-trait-pair tests). coloc.abf derives the posterior probabilities directly from the marginal association statistics (per-variant effect sizes and standard errors) under a single-causal-variant assumption and requires no LD reference panel. Prior probabilities: p1 = p2 = 1x10^-4 and p12 = 5x10^-6 (half of the coloc default of 1x10^-5, set conservatively to reduce false-positive colocalization; Wallace 2020). PP.H0-PP.H4: posterior probabilities for no association (H0), association with PD only (H1), the kidney trait only (H2), both traits with distinct causal variants (H3), or a single shared causal variant (H4). sum_H3H4: PP.H3 + PP.H4. ratio_H4/H3: PP.H4/PP.H3. classification: 'shared (PP.H4>0.80)', a stringent shared-causal-variant call; 'shared (composite criterion)', PP.H3+PP.H4 >= 0.8 and PP.H4/PP.H3 >= 5 without reaching PP.H4>0.80; 'distinct (PP.H3>0.80)'; or 'inconclusive'. genomic_locus: identifier grouping locus-trait-pair tests whose lead variants fall within 500 kb on the same chromosome into a single genomic locus; the 175 tests collapse to 84 distinct non-MHC genomic loci, of which 4 show a shared causal variant (PP.H4>0.80), a 5th meets the composite criterion, 12 show distinct causal variants (PP.H3>0.80), and 67 are inconclusive. lead_SNP: conjFDR lead variant. candidate_shared_variant: variant with the highest posterior probability of being the shared causal variant (SNP.PP.H4) at colocalized loci.

n_shared_snps: variants present in both GWAS within the +/-500 kb window. The eGFRcys trait pair is the cystatin C-based sensitivity/replication analysis (Stanzick et al., 2021); MHC (chr6:25-34 Mb) was excluded.

Supplementary Data 13. **Brain cis-eQTL annotation of the candidate shared variant at each colocalized locus (GTEx v11)**. Significant cis-eQTL associations in GTEx v11 brain tissues for the candidate shared variant at each colocalized locus. At each locus showing colocalization between Parkinson's disease (PD) and a kidney-related trait, the variant with the highest posterior probability of being the shared causal variant (maximum per-SNP colocalization probability, SNP.PP.H4) was queried against GTEx v11, and all significant variant-gene pairs across brain regions are reported. Each row is a single variant-gene-tissue association; a variant therefore appears in multiple rows where it is an eQTL for several genes or across several regions. Locus (candidate variant), the colocalized locus and the candidate shared variant queried; results are shown for the three colocalized loci with a GTEx signal (TNK2/rs13059257, BIN3/rs13264187, MSRA-PRSS51/rs6982308). The RAB19 locus (candidate variant rs12216582) is omitted because it returned no significant GTEx cis-eQTL. Gencode Id, Ensembl/GENCODE gene identifier; Gene Symbol, HGNC gene symbol (the Ensembl identifier is shown where no approved symbol exists); Variant Id, GRCh38 variant identifier (chromosome_position_reference_alternative_build); SNP Id, dbSNP reference SNP identifier; P-Value, nominal cis-eQTL association P value; NES, normalized effect size (the eQTL slope, for the alternative relative to the reference allele); Tissue, GTEx brain region.

Supplementary Data 14. **Glomerular and tubulointerstitial cis-eQTL annotation of the candidate shared variant at each colocalized locus (NephQTL2)**. Significant glomerular and tubulointerstitial cis-eQTL associations in NephQTL2 (NEPTUNE cohort) for the candidate shared variant at each colocalized locus. Each candidate shared variant (maximum SNP.PP.H4) was queried against both the glomerular and the tubulointerstitial compartments, and genes that are significant eGenes (gene-level FDR < 0.05) are reported. Locus (candidate variant), colocalized locus and variant queried (TNK2/rs13059257, BIN3/rs13264187, MSRA-PRSS51/rs6982308, RAB19/rs12216582); Compartment, glomerular or tubulointerstitial; rsid, snp_hg19, Variant ID, candidate variant identifiers; Gene symbol, HGNC gene symbol; Gene, Ensembl gene identifier; Entrez, Entrez Gene identifier; Gene Type, Ensembl gene biotype; Gene-level FDR, false-discovery rate for the gene being an eGene; eGene, significant-eGene indicator (1); Model Size, number of variants in the eGene model; SNP Beta, SNP P-Value, cis-eQTL effect size and association P value for the candidate variant itself; candidate_variant_cis-eSNP, whether the candidate variant is itself a significant cis-eSNP for the eGene ('Bonferroni (P<8.3x10^-4)', 'nominal (P<0.05)', or 'no'). Single-nucleus expression columns give expression of each eGene across cell types: the glomerular panel comprises podocytes (POD), parietal epithelial cells (PEC), mesangial cells (MES), fibroblasts (FIB), endothelial cells (ENDO) and leukocytes (LEUK); the tubulointerstitial panel comprises proximal tubule (PT), principal cells (PC), type-A and type-B intercalated cells (ICA, ICB), distal convoluted tubule (DCT), connecting tubule (CNT), thick ascending limb (TAL), endothelial cells (ENDO) and leukocytes (LEUK);

cell types not applicable to a compartment are left blank. Notably, the BIN3 candidate variant rs13264187 is a significant cis-eSNP for the podocyte-expressed senescence regulator CCAR2 in both compartments (glomerular SNP P = 4.4x10^-19, tubulointerstitial SNP P = 3.0x10^-13).

Supplementary Data 15. **Gene-set enrichment analysis from FUMA GENE2FUNC**. Gene-set enrichment was performed using FUMA GENE2FUNC on genes mapped to conjFDR-identified shared loci. P-values were corrected using the Benjamini-Hochberg false discovery rate (FDR < 0.05). N_genes: total genes in the gene set. N_overlap: number of mapped genes overlapping the gene set.

Supplementary Data 16. **Network-based gene-set enrichment analysis from NetworkAnalyst**. Protein-protein interaction (PPI) network analysis was performed using NetworkAnalyst. Genes mapped to conjFDR-identified shared loci were used as input for each PD–kidney trait pair. Enrichment was tested across KEGG pathways, Reactome pathways, Gene Ontology Biological Process (GOBP), and Gene Ontology Molecular Function (GOMF). P-values were corrected using the Benjamini-Hochberg false discovery rate (FDR < 0.05). Total: number of genes in the pathway. Expected: expected number of overlapping genes by chance. Hits: observed number of overlapping genes. Genes: overlapping gene symbols.

Supplementary Data 17. **Local genetic correlations between Parkinson's disease and kidney-related traits using LAVA**. Local genetic correlations were estimated using LAVA at genomic loci where both traits exhibited significant local heritability (univariate P < 0.05/2,495). Locus: LAVA LD block identifier. n_snps: number of SNPs in the locus. n_pcs: number of principal components retained after singular value decomposition (accounting for ≥99% of variance). rho: local genetic correlation, ranging from −1 to +1. rho.lower, rho.upper: 95% confidence interval for ρ. r^2: proportion of local genetic variance in one trait explained by the other. p: simulation-based P-value.

Supplementary Data 18. **conjunctional FDR results for SNPs reaching conjFDR < 0.05 for PD (sex-combined) x cystatin C-based eGFR (eGFRcys; sex-combined)**. conjunctional FDR (conjFDR) results for the cystatin C-based eGFR (eGFRcys) sensitivity/replication analysis (PD, sex-combined x eGFRcys). Because creatinine-based eGFR partly reflects skeletal muscle mass, the PD-eGFR analysis was repeated with eGFRcys, a marker largely independent of muscle mass (Stanzick et al., 2021; n = 460,826), addressing potential muscle-mass confounding and providing a lower-powered replication. Columns: locusnum, conjFDR locus number (SNPs grouped into physically contiguous loci); rsid, dbSNP reference SNP ID; chrnum and chrpos, chromosome and base-pair position (GRCh37); zscore_eGFRcys and zscore_PD, GWAS z-scores; conjfdr_* , conjunctional FDR; prune_*, random-pruning indicator (1 marks the SNP retained as an LD-independent representative after random pruning, 0 otherwise); min_conjfdr, the minimum conjFDR value (identical to the conjfdr column for all SNPs reported here); pval_* columns, two-sided GWAS association p-values for the two traits. Column names carry the specific stratum/trait labels of this pair. Where present, MHC

loci (chr6:25-34 Mb, GRCh37) are reported here but excluded from all downstream (colocalization and enrichment) analyses.

Supplementary Data 19. **conjFDR threshold-sensitivity analysis for PD (sex-combined) × creatinine-based eGFR**. conjFDR threshold-sensitivity analysis. The number of independent conjFDR loci retained at progressively stricter thresholds (< 0.05, < 0.01, < 0.001) is reported for non-MHC loci and for all loci including the 3 MHC loci, together with the identity of the loci retained at conjFDR < 0.001. The core shared PD-eGFR loci (FAM47E-STBD1/SCARB2, CTSB, TMEM163) persist under stricter thresholds, demonstrating that the PD-eGFR conjFDR signal is not an artifact of the permissiveness of the conjFDR < 0.05 criterion.

Supplementary Data 20. **Prior-sensitivity of the coloc.abf colocalization analysis**. Colocalization posterior probabilities (PP.H0–PP.H4), the PP.H4/PP.H3 evidence ratio, and a classification for each colocalized locus and the SCARB2/FAM47E-STBD1 distinct-variant control, computed with coloc.abf across a grid of priors: $p12 = 1\times10^{-6}$, 5×10^{-6} (primary), 1×10^{-5} (the coloc default) and 5×10^{-5} at $p1 = p2 = 1\times10^{-4}$, and a less conservative setting ($p1 = p2 = 1\times10^{-3}$, $p12 = 1\times10^{-4}$). PP.H4 = single shared causal variant; PP.H3 = distinct causal variants; PP.H4/PP.H3 = evidence ratio favouring a shared over a distinct variant. classification: shared (PP.H4 > 0.80); leans shared (PP.H4 > PP.H3 but below 0.80); or distinct (PP.H3 > 0.80). All shared loci remain shared (PP.H4 > 0.80) across the primary prior and every less conservative prior including the coloc default; RAB19 remains shared across the entire grid; the SCARB2/FAM47E-STBD1 control remains distinct (PP.H3 > 0.999) under every prior. At every locus and prior PP.H4 exceeds PP.H3, so the evidence favours a shared over a distinct causal variant throughout; only under the stringent $p12 = 1\times10^{-6}$ do the more borderline loci fall below the 0.80 confidence threshold, and even then the posterior mass shifts to the null and single-trait hypotheses (H0–H2) rather than to a distinct-variant model (H3) — the prior affects the confidence, not the direction, of the colocalization calls. Same ±500 kb window, QC and input data as Supplementary Table 12.

Supplementary Data 21. **SLALOM (DENTIST-S) LD-consistency QC of the reported colocalized loci**. SLALOM (Kanai et al., 2022) DENTIST-S diagnostic applied to every reported colocalized locus and the SCARB2/FAM47E-STBD1 distinct-variant control, using a custom in-sample UK Biobank LD reference (N = 337,462 unrelated white-British individuals). For each trait at each locus, SLALOM tests whether each variant's association statistic is consistent with the lead variant given local LD (a simplified DENTIST test; Chen et al., 2021); a DENTIST-S outlier has $r^2 > 0.6$ to the lead and DENTIST-S $P < 1\times10^{-4}$, and a locus with zero outliers is not suspicious for meta-analysis fine-mapping. Columns: lead SNP (rsID) = the conjFDR lead (sentinel) variant at the locus; variants tested (r^2>0.6) = array variants in LD with the lead evaluated; DENTIST-S outliers = number flagged; max PIP = maximum approximate-Bayes-factor posterior inclusion probability at the locus; verdict = consistent (0 outliers) or flagged. All 18 colocalized locus–trait tests showed zero outliers; the single flag (SCARB2/eGFR, chr4:77,360,431) has PIP ≈ 0 and is neither the lead nor in the 95% credible set (DENTIST-S $-\log_{10}p = 4.3$), and SCARB2 is a distinct-variant locus, not a

colocalization claim. The LD reference was used only to diagnose summary-statistic/LD inconsistency (following Kanai et al.).
