# Supplementary material for: Genome-wide cross-trait analysis to compare genetic architecture between Parkinson’s disease and kidney-related traits
Source: Nat Commun. 2026 Sep 18;17:10966. doi: 10.1038/s41467-026-77920-3 (PMC13683393; doi:10.1038/s41467-026-77920-3)
Supplement: Supplementary file 3 [file 41467_2026_77920_MOESM4_ESM.pdf]

nature portfolio

Corresponding author(s): Sarah A Gagliano Taliun

Last updated by author(s): Sep 1, 2026

Reporting Summary

Nature Portfolio wishes to improve the reproducibility of the work that we publish. This form provides structure for consistency and transparency in reporting. For further information on Nature Portfolio policies, see our Editorial Policies and the Editorial Policy Checklist.

Statistics

For all statistical analyses, confirm that the following items are present in the figure legend, table legend, main text, or Methods section.

n/a	Confirmed	
☐	☒	The exact sample size (*n*) for each experimental group/condition, given as a discrete number and unit of measurement
☒	☐	A statement on whether measurements were taken from distinct samples or whether the same sample was measured repeatedly
☒	☐	The statistical test(s) used AND whether they are one- or two-sided<br>*Only common tests should be described solely by name; describe more complex techniques in the Methods section.*
☒	☐	A description of all covariates tested
☐	☒	A description of any assumptions or corrections, such as tests of normality and adjustment for multiple comparisons
☒	☐	A full description of the statistical parameters including central tendency (e.g. means) or other basic estimates (e.g. regression coefficient) AND variation (e.g. standard deviation) or associated estimates of uncertainty (e.g. confidence intervals)
☒	☐	For null hypothesis testing, the test statistic (e.g. *F*, *t*, *r*) with confidence intervals, effect sizes, degrees of freedom and *P* value noted<br>*Give P values as exact values whenever suitable.*
☒	☐	For Bayesian analysis, information on the choice of priors and Markov chain Monte Carlo settings
☒	☐	For hierarchical and complex designs, identification of the appropriate level for tests and full reporting of outcomes
☐	☒	Estimates of effect sizes (e.g. Cohen's *d*, Pearson's *r*), indicating how they were calculated

Our web collection on statistics for biologists contains articles on many of the points above.

Software and code

Policy information about availability of computer code

Data collection	This study analysed genome-wide association study summary statistics and individual-level UK Biobank data; no new participant data were collected.
Data analysis	The code used for this work is available on GitHub: https://github.com/GaglianoTaliun-Lab/kidney-neurodegen ((https://doi.org/10.5281/zenodo.22150293).

For manuscripts utilizing custom algorithms or software that are central to the research but not yet described in published literature, software must be made available to editors and reviewers. We strongly encourage code deposition in a community repository (e.g. GitHub). See the Nature Portfolio guidelines for submitting code & software for further information.

Data

Policy information about availability of data

All manuscripts must include a data availability statement. This statement should provide the following information, where applicable:

- Accession codes, unique identifiers, or web links for publicly available datasets
- A description of any restrictions on data availability
- For clinical datasets or third party data, please ensure that the statement adheres to our policy

This study analysed genome-wide association study summary statistics and individual-level UK Biobank data; no new participant data were collected. Sex-stratified Parkinson’s disease summary statistics (Blauwendraat et al.) are available at https://personal.broadinstitute.org/ryank/Blauwendraat_Parkinsons_sexdiff_2021.zip. Sex-combined estimated glomerular filtration rate summary statistics from the CKDGen Consortium and UK Biobank meta-analysis (Stanzick et al.) are available at

https://ckdgen.imbi.uni-freiburg.de/. Genome-wide sex-specific summary statistics for hematuria are available at https://my.locuszoom.org/gwas/496796/ (female-specific) and https://my.locuszoom.org/gwas/494413/ (male-specific). Sex-combined summary statistics for hematuria are available at https://pheweb.org/UKB-SAIGE/pheno/593. The sex-stratified estimated glomerular filtration rate, urinary albumin-to-creatinine ratio, urinary potassium-to-creatinine ratio, urinary sodium-to-creatinine ratio summary statistics generated in this study from the UK Biobank have been deposited in Zenodo https://doi.org/10.5281/zenodo.22150292. UK Biobank individual-level data are available under restricted access because access is governed by the UK Biobank Access Management System; access can be obtained by submitting a research application at https://www.ukbiobank.ac.uk/enable-your-research/apply-for-access. Expression quantitative trait locus data were obtained from GTEx v11 (https://gtexportal.org/home/downloads/adult-gtex) and NephQTL2 (https://nephqtl2.org). Linkage disequilibrium reference data were obtained from the 1000 Genomes Project Phase 3 European reference panel (https://www.internationalgenome.org/data). Gene sets were queried through FUMA v 2.1.6 (https://fuma.ctglab.nl) and NetworkAnalyst 3.0 (https://www.networkanalyst.ca).

Research involving human participants, their data, or biological material

Policy information about studies with human participants or human data. See also policy information about sex, gender (identity/presentation), and sexual orientation and race, ethnicity and racism.

Reporting on sex and gender	Gender was not considered. Summary-level genome-wide association study summary statistics either assessed both males and females or were sex-specific.
Reporting on race, ethnicity, or other socially relevant groupings	Race, ethnicity or other socially relevant groupings were not considered.
Population characteristics	Summary-level genome-wide association study data were used, described in Table 1.
Recruitment	Not applicable as we used existing summary-level genome-wide association study statistics and data from UK Biobank.
Ethics oversight	The UK Biobank has ethical approval from the North West Multi-centre Research Ethics Committee, and all UK Biobank participants provided written informed consent. Researchers can operate under this approval with no need for further ethical approval.

Note that full information on the approval of the study protocol must also be provided in the manuscript.

Field-specific reporting

Please select the one below that is the best fit for your research. If you are not sure, read the appropriate sections before making your selection.

[x] Life sciences [ ] Behavioural & social sciences [ ] Ecological, evolutionary & environmental sciences

For a reference copy of the document with all sections, see nature.com/documents/nr-reporting-summary-flat.pdf

Life sciences study design

All studies must disclose on these points even when the disclosure is negative.

Sample size	The sample size of each genome-wide association study summary statistics file is provided in Table 1.
Data exclusions	The summary statistics were derived from analyses carried out in individuals of European genetic ancestry. We acknolwedge this choice as a limitation in the discussion.
Replication	Not relevant. Existing summary-level genome-wide association study data were used.
Randomization	Not relevant. Existing summary-level genome-wide association study data were used.
Blinding	Not relevant. Existing summary-level genome-wide association study data were used.

Reporting for specific materials, systems and methods

We require information from authors about some types of materials, experimental systems and methods used in many studies. Here, indicate whether each material, system or method listed is relevant to your study. If you are not sure if a list item applies to your research, read the appropriate section before selecting a response.

Materials & experimental systems

n/a	Involved in the study
☒	☐ Antibodies
☒	☐ Eukaryotic cell lines
☒	☐ Palaeontology and archaeology
☒	☐ Animals and other organisms
☒	☐ Clinical data
☒	☐ Dual use research of concern
☒	☐ Plants

Methods

n/a	Involved in the study
☒	☐ ChIP-seq
☒	☐ Flow cytometry
☒	☐ MRI-based neuroimaging

Plants

Seed stocks	*Report on the source of all seed stocks or other plant material used. If applicable, state the seed stock centre and catalogue number. If plant specimens were collected from the field, describe the collection location, date and sampling procedures.*
Novel plant genotypes	*Describe the methods by which all novel plant genotypes were produced. This includes those generated by transgenic approaches, gene editing, chemical/radiation-based mutagenesis and hybridization. For transgenic lines, describe the transformation method, the number of independent lines analyzed and the generation upon which experiments were performed. For gene-edited lines, describe the editor used, the endogenous sequence targeted for editing, the targeting guide RNA sequence (if applicable) and how the editor was applied.*
Authentication	*Describe any authentication procedures for each seed stock used or novel genotype generated. Describe any experiments used to assess the effect of a mutation and, where applicable, how potential secondary effects (e.g. second site T-DNA insertions, mosiacism, off-target gene editing) were examined.*
