# Supplementary material for: Genome-wide cross-trait analysis to compare genetic architecture between Parkinson’s disease and kidney-related traits
Source: Nat Commun. 2026 Sep 18;17:10966. doi: 10.1038/s41467-026-77920-3 (PMC13683393; doi:10.1038/s41467-026-77920-3)
Supplement: Supplementary file 4 [file 41467_2026_77920_MOESM5_ESM.pdf]

nature portfolio

Peer Review File

Genome-wide cross-trait analysis to compare genetic architecture between Parkinson's disease and kidney-related traits

Corresponding Author: Dr Sarah Gagliano Taliun

This file contains all reviewer reports in order by version, followed by all author rebuttals in order by version.

Version 0:

Reviewer comments:

Reviewer #1

(Remarks to the Author)
Dr. Gawhary and colleagues propose an investigation on the common genetic roots of kidney function traits and AD and PD. The work is clearly presented and well conducted. I strongly appreciated the running of analyses after removing proxy cases. I would invite the Authors to consider the following issues:

1. "Kidney" phenotypes:

a. A set of urinary traits and a no-better specified eGFR are indicated as kidney traits. Please, define these traits better: what do they represent? The fact that something is excreted through the urine doesn't automatically implicate kidney function. For instance, circulating sodium/potassium balance is key to maintain cardiac and brain functions. The balance is difficult to be maintained at older ages, often resulting in apparent dementia. Please, try to focus on the specific traits and outline more contextualized discussion on their relation with PD and/or AD.

b. In the Methods „Genome-wide association study (GWAS) summary statistics" section, only PD and AD but not urinary and kidney GWAS are described. Please, add description of the latter.

c. Please, define which measure of eGFR was used: was it estimated based on serum creatinine, serum cystatin C, or both? Which formula was used (CKD-EPI, race-free CKD-EPI, …)? In particular, in addition to kidney function, serum creatinine could reflect muscle mass metabolism. Given muscle weakness is typical of the elderly, which are also more affected by PD and AD, it is important to provide evidence based on an alternative marker, such as cystatin C, or directly using an eGFR estimation based on both creatinine and cystatin C. PMID: 39927731 gives relevant insights into this issue.

d. Urinary traits: please, run an additional sensitivity analysis adjusting by urinary creatinine. Even if the correct biomarker levels should be obtained using urine creatinine as the denominator, caution is needed when using ratios as GWAS outcomes (PMID: 39818621).

e. Given CKD is extensively mentioned in the Introduction, why didn't the Authors consider running analyses on CKD?

2. While the introduction depicted quite well the research question on finding the common roots of kidney dysfunction and PD/AD, in the Results and Discussion, this key aspect is not outlined very well. I got lost in a series of statement about inclusion/exclusion of proxy cases, and about sex stratification. Those are very interesting points, but they were not the main reason why this study was conducted. In the end: do kidney function and AD/PD share substantial genetic correlation? What are the molecular pathways that are mostly responsible for the observed correlation? These aspects are present, but please, try to reorganize the Results and Discussion to make them clearer.

3. Ageing and survival bias

a. Please, outline the age range of the different GWAS used in this study

b. How does the different age range affects the estimates? I guess GWAS of the kidney traits were conducted on people of any age above 18 years, while AD/PD GWAS were ran in 60+ year-old individuals

c. I think the risk of sample selection should be recognized. Both the kidney and the brain deteriorate with age. I wonder whether there could be collider or survival biases affecting the analyses and how this could be prevented. Calibrating GWAS results to the reference population (least for the kidney part) might be a valuable sensitivity analysis to conduct (PMID: 40234401; PMID: 37106081)

4. Suppl. Tab. 1 should be moved to the main manuscript (maybe including the information in Fig. 1 or as a separate table). Please, add the source of the GWAS.

5. Prior to entering the genetic correlation analyses, where GWAS summary statistics corrected for genomic inflation?

6. Given the work presents many levels of analysis (with/without proxy cases; sex-stratification), I would recommend to avoid running both strict and lenient gene-set enrichment analysis with LAVA: please, limit to “strict”.

7. Line 352: please, avoid using “etc.” when presenting results.

8. Line 359: a “.” is missing after the parenthesis

Reviewer #2

(Remarks to the Author)
Gawhary et al. present global and local genetic correlation analyses between kidney-related traits and late-onset neurodegenerative diseases (Alzheimer's disease and Parkinson's disease). They collected large-scale publicly available summary statistics, with and without inclusion of proxy cases or sex-stratification. While statistical power for AD is limited, they found several significant global genetic correlations between kidney-related traits and PD. Local genetic correlation, gene-set enrichment, and network-based enrichment analyses were conducted, but limited mechanistic insights are provided beyond the number of significant results.

While the manuscript is of interest and could be one of the first studies to systematically examine genetic correlations between kidney-related traits and neurodegenerative diseases, there are a couple of major concerns.

1. Local genetic correlation results severely lack interpretability. One of the major advantages of local genetic correlation analysis over global genetic correlation is that it allows investigation of which genetic variants contribute to shared genetic architecture between traits. Currently, the manuscript only presents the numbers and fractions of overlaps, and buries the majority of the results in numerous Supplementary Tables or Figures. To mechanistically interpret the shared local genetic architecture between kidney-related traits and neurodegenerative disorders, the authors should provide a detailed investigation of why two traits show significant local genetic correlation.
2. Similarly, only limited results are provided for gene-set enrichment or network-based enrichment analyses in the main text. Only a few loci, such as NR1H2/NR1H3 for AD and metabolism, are documented in the text, with limited description of their potential implications or relevance.
3. Main figures only cover global genetic correlation analyses. It is disappointing to see that the figures are only heatmaps of global genetic correlation and that none of the local genetic correlation, gene-set enrichment, or network-based enrichment analyses are represented in the main figures.

Reviewer #3

(Remarks to the Author)
This study investigates genetic correlations between six kidney-related traits and two late-onset neurodegenerative diseases (Alzheimer’s and Parkinson’s). Using GWAS data, the authors estimate both global and local genetic correlations, analyzing the influence of proxy cases and sex-stratification. Significant correlations were identified for Parkinson’s disease with urinary sodium (Rg=-0.17) and urinary creatinine (Rg=-0.16) levels. Enrichment analyses point to shared immune, metabolic, and viral response pathways. The findings support a kidney-brain axis and emphasize the importance of considering sex differences and proxy inclusion in genetic studies.

The analytical rigor, stratification by sex, and attention to proxy inclusion are commendable. However, important limitations in phenotype definition, particularly for urinary biomarkers, require major revision, as they impact the interpretation of the observed associations.

The study identifies significant genetic correlations between Parkinson’s disease and urinary sodium and urinary creatinine. However, the lack of correction for urine dilution (e.g., using urinary creatinine as a denominator or normalization to osmolarity) introduces major interpretative uncertainty. Urinary sodium in spot samples is highly sensitive to hydration status and recent dietary salt intake, and without normalization (e.g., uNa/uCr), may reflect extrarenal factors rather than kidney-specific function. Urinary creatinine itself is a function of muscle mass, hydration, and tubular handling, complicating its interpretation as a standalone phenotype. Lastly, diuretic use, common in aging populations and in PD in particular (due to comorbidities like hypertension), can dramatically alter urinary sodium levels.

Given these limitations, the observed genetic correlations may reflect shared genetic influences on systemic volume regulation, salt handling, or hydration state, rather than intrinsic renal function per se.

To strengthen the biological interpretation of the findings, it is necessary to recompute key urinary phenotypes with dilution correction, ideally as ratios to urinary creatinine (e.g., uNa/uCr or UACR). It would further help to exclude or stratify by diuretic use. Such refinements would help disentangle kidney-specific effects from broader homeostatic or external influences, and provide a more reliable readout of kidney–brain genetic correlation.

In addition, for phenotypes showing significant local genetic correlation, colocalization analyses could help clarify whether observed overlaps are driven by shared causal variants. This would complement the LAVA results and provide further insight into potential mechanistic links between kidney traits and neurodegeneration.

In summary, this is a well-designed and timely study with high potential impact. To substantially improve the validity of the results, it is essential to address urinary dilution correction. Furthermore, we recommend extending the analysis with colocalization studies to enhance interpretability and translational value.

Version 1:

Reviewer comments:

Reviewer #1

(Remarks to the Author)
I'd like to thank the Authors for their revision of the manuscript. However, my main concerns remained only partially addressed. In my previous report, I mentioned several potential risks of confounding. The most important one is that creatinine-based eGFR partly reflects muscle mass metabolism. Muscle mass changes with age, along with the risk of PD. For instance, cellular senescence might affect both PD risk and creatinine levels through muscle mass changes, without necessarily going through kidney dysfunction. At minimum, this risk should be acknowledged in the Discussion. In addition, GWAS of cystatin C do exist and I think Authors could attempt a sensitivity analysis (to avoid disrupting the current flow of presentation).

Reviewer #2

(Remarks to the Author)
I thank the authors for their extensive revision. In response to the review panel, they have narrowed the scope to PD and revised their methodological approach, adopting conjFDR and colocalization with SuSiE fine-mapping in place of LAVA. My second and third comments from the first round are largely addressed.

While the manuscript is now more focused and the analytical toolkit appears more modern, I have several major concerns regarding the revised approach and its interpretation.

1. Colocalization and SuSiE fine-mapping of meta-analysis GWAS with an external LD reference panel are not reliable.

In the revised pipeline, the authors use coloc.susie (Wallace et al. 2021) to perform colocalization with SuSiE fine-mapping. This approach has serious methodological flaws.

a) Multiple-causal-variant fine-mapping with SuSiE requires accurate in-sample LD. Substituting an external LD reference (1000 Genomes Phase 3 EUR, with only ~500 samples) is strictly not recommended and produces many false positives (Ulirsch et al. 2019; Weissbrod et al. 2020).

b) Meta-analysis makes this worse. Both input GWAS are themselves meta-analyses: the sex-combined PD sumstats are a meta-analysis of the sex-stratified datasets, and the sex-combined eGFR sumstats are a meta-analysis of CKDGen and UKB. This is precisely the setting in which the 61-locus PD-eGFR signal and all ten "shared" colocalized loci arise. Beyond the absence of in-sample LD, fine-mapping and colocalization of meta-analysis sumstats are known to be miscalibrated and error-prone (Kanai et al. 2022).

These issues are the most likely reason SuSiE failed to converge at the majority of loci (only 10 converged). The coloc.abf fallback does not resolve the problem, because it relies on single-causal-variant ABF fine-mapping, and the meta-analysis miscalibration still applies.

Given these severe flaws, the fine-mapping and colocalization results presented in this study cannot be considered reliable.

2. Even taking the colocalization results at face value, the central "shared architecture" framing is not well supported.

Across 128 non-MHC loci, distinct causal variants (PP.H3 > 0.8, n = 40) outnumber shared causal variants (PP.H4 > 0.8, n = 10). The dominant empirical result is therefore that PD and kidney-trait signals colocalize to the same regions but are driven

by distinct variants. The Discussion acknowledges this, yet the title, abstract, and summary statements continue to lead with "shared genetic architecture." I ask the authors to recalibrate the framing throughout to reflect the predominance of distinct causal variants, reserving the shared-variant interpretation for the minority of loci, concentrated in PD-eGFR, that genuinely meet the colocalization criteria.

3. The robustness of the conjFDR results should be established.

The original manuscript's central claims rested on LAVA local genetic correlations. Once these became non-significant under the recommended UK Biobank LD reference panel, the authors replaced LAVA with conjFDR as the primary locus-discovery method, without explicitly justifying this substitution. Because the revised LAVA analysis is now completely null, the new conjFDR results require independent support to reconcile these apparently contradictory findings.

The number of shared loci tracks the statistical power of the kidney GWAS: PD-eGFR yields 61 loci, whereas the smaller kidney GWAS yield between 0 and 11. Because conjFDR < 0.05 is a permissive criterion and is sensitive to differential power between the two input datasets, the possibility that the PD-eGFR signal is inflated by the exceptional power of the eGFR GWAS should be addressed directly, for example through threshold-sensitivity analysis or a down-sampling or power-matched comparison.

Reviewer #3

(Remarks to the Author)
Thank you very much for addressing my questions and concerns. I believe the revisions have substantially strengthened the manuscript.

Version 2:

Reviewer comments:

Reviewer #1

(Remarks to the Author)
I'd like to thank the Authors for addressing my concerns. I have no further questions.

Reviewer #2

(Remarks to the Author)
I thank the authors for their extensive revision. The revised manuscript addressed the majority of my concerns, including the revised colocalization methods and the recalibrated title and abstract.

I only have one minor comment. While the authors claimed the colocalizations “were stable across a grid of priors”, ST20 showed PP.H4 <= 0.80 at p12 = 1e-6 (stringent) for four of the five shared calls. Minor limitation texts would suffice.

Open Access This Peer Review File is licensed under a Creative Commons Attribution 4.0 International License, which permits use, sharing, adaptation, distribution and reproduction in any medium or format, as long as you give appropriate credit to the original author(s) and the source, provide a link to the Creative Commons license, and indicate if changes were made.
In cases where reviewers are anonymous, credit should be given to 'Anonymous Referee' and the source.
The images or other third party material in this Peer Review File are included in the article's Creative Commons license, unless indicated otherwise in a credit line to the material. If material is not included in the article's Creative Commons license and your intended use is not permitted by statutory regulation or exceeds the permitted use, you will need to obtain permission directly from the copyright holder.
To view a copy of this license, visit https://creativecommons.org/licenses/by/4.0/

We thank the reviewers for reviewing our manuscript. We outline our responses to the individual comments in bold below. For your reference, we include a tracked-changes version of the manuscript as well as a clean version. In summary, we have completely reperformed analyses and rewritten the manuscript based on the reviewers' comments as well as recent methodological recommendations for performing local genetic correlation analyses using LAVA in European genetic ancestry datasets. In light of these changes, we have chosen to narrow the scope to assess only PD (rather than also AD) to better focus the paper on the possible biological mechanisms linking PD with kidney-related traits.

Reviewer #1

Dr. Gawhary and colleagues propose an investigation on the common genetic roots of kidney function traits and AD and PD. The work is clearly presented and well conducted. I strongly appreciated the running of analyses after removing proxy cases. I would invite the Authors to consider the following issues:
We thank the reviewer for their attentive review and comments.

1. "Kidney" phenotypes:

a. A set of urinary traits and a no-better specified eGFR are indicated as kidney traits. Please, define these traits better: what do they represent? The fact that something is excreted through the urine doesn't automatically implicate kidney function. For instance, circulating sodium/potassium balance is key to maintain cardiac and brain functions. The balance is difficult to be maintained at older ages, often resulting in apparent dementia. Please, try to focus on the specific traits and outline more contextualized discussion on their relation with PD and/or AD.
Thank you for this comment. We have now re-performed analyses using generally accepted indicators of kidney function, specifically: eGFR (creatinine-based estimation of glomerular filtration), uACR (measure of protein in the urine), hematuria (measure of blood in the urine), urinary sodium levels adjusted for urinary creatinine and urinary potassium levels adjusted for urinary creatinine. Given the substantial reframing of this manuscript (see responses to comments below), we have focused the paper on PD and the kidney-related metrics. We have contextualized discussion on their relationship with PD.

b. In the Methods „Genome-wide association study (GWAS) summary statistics" section, only PD and AD but not urinary and kidney GWAS are described. Please, add description of the latter.
A description of all the kidney GWAS has now been added to the Methods. Additionally, we have substantially re-worked the table summarizing key components of all the GWAS used in this study for added clarity: the new Table 1.

c. Please, define which measure of eGFR was used: was it estimated based on serum creatinine, serum cystatin C, or both? Which formula was used (CKD-EPI, race-free CKD-EPI, …)? In particular, in addition to kidney function, serum creatinine could reflect muscle mass metabolism. Given muscle weakness is typical of the elderly, which are also more affected by PD and AD, it is important to provide evidence based on an alternative marker, such as cystatin C, or directly using an eGFR estimation based on both creatinine and cystatin C. PMID: 39927731 gives relevant insights into this issue.
eGFR based on serum creatinine was used. Specifically, for the eGFR sex-combined GWAS carried out by Stanzick et al., was estimated using the Chronic Kidney Disease Epidemiology Collaboration (CKD-EPI 2009) formula. Adjustment for 'race' was not relevant as the GWAS focused on European ancestry individuals.

d. Urinary traits: please, run an additional sensitivity analysis adjusting by urinary creatinine. Even if the correct biomarker levels should be obtained using urine creatinine as the denominator, caution is needed when using ratios as GWAS outcomes (PMID: 39818621).
Thank you. We have adjusted our choice of kidney traits accordingly using urinary creatinine in the denominator (a point that was also brought up by Reviewer 3); please refer to point 1a above. The Methods have been updated to reflect these changes, and we have rerun all analyses based on this updated set of kidney traits.

e. Given CKD is extensively mentioned in the Introduction, why didn't the Authors consider running analyses on CKD?
The latest and largest GWAS studies focus on commonly-used quantitative/continuous metrics that can be indicative of CKD (namely, eGFR, e.g. Stanzick et al. 2021; https://doi.org/10.1038/s41467-021-24491-0) as opposed to the binary case/control setting. The reason is largely due to statistical power, whereby a GWAS for a quantitative trait has more statistical power.

2. While the introduction depicted quite well the research question on finding the common roots of kidney dysfunction and PD/AD, in the Results and Discussion, this key aspect is not outlined very well. I got lost in a series of statement about inclusion/exclusion of proxy cases, and about sex stratification. Those are very interesting points, but they were not the main reason why this study was conducted. In the end: do kidney function and AD/PD share substantial genetic correlation? What are the molecular pathways that are mostly responsible for the observed correlation? These aspects are present, but please, try to reorganize the Results and Discussion to make them clearer.
Now, by restricting our kidney phenotypes to those that have clear relevance to kidney function (eGFR, uACR and hematuria), we have significantly streamlined the results (and supplements) to maintain the focus in the Introduction throughout on the potential biological implications influencing kidney and brain health by decreasing the focus on the

methodological aspects (e.g. inclusion/exclusion of proxy cases and sex stratification) in the results.

The developers of the LAVA software have since strongly recommended the use of UK Biobank instead of 1000 Genomes for local genetic correlation analyses in European ancestry datasets. Given this new methodological insight, we have now re-run all LAVA analyses following this recommendation. Additionally, as a complementary approach to test for potential shared genetics at particular loci, we have now also used the conjFDR tool, which is commonly done in the field to investigate potentially shared loci from a different methodological perspective (i.e. not based on genetic correlation).

3. Ageing and survival bias
a. Please, outline the age range of the different GWAS used in this study
Almost all of the kidney-related traits GWAS assessed were conducted in the UK Biobank cohort (age range 40-69 years). The exception is sex-combined eGFR, which was a meta-analysis between the UK Biobank as well as from the CKDGen Consortium. The latter is composed of adults (18+ years), including those younger than the UK Biobank participants; nevertheless, the median age of CKDGen partcipants, as previously described is in line with the UK Biobank age range at baseline: 54 years (Wuttke et al. PMID: 31152163).

As for the Parkinson's GWAS (Blauwendraat et al.), controls were participants with neither reported PD nor a parent with PD, and with an age of recruitment over 60 years old. Analyses carried out by Blauwendraat et al. were adjusted for age, specifically age at onset for cases and age of last examination for controls.

b. How does the different age range affects the estimates? I guess GWAS of the kidney traits were conducted on people of any age above 18 years, while AD/PD GWAS were ran in 60+ year-old individuals
As noted in the, we now only focus on the relationship between the tested kidney traits and PD given the availability of both sex-combined and sex-stratified data, which is a key scientific question that our paper aims to address.

As we now note in the Methods, age was included as a covariate for all the kidney-related GWAS analyses.

As for the GWAS for PD, when age information is provided, the cases (as the reviewer notes do tend to be older individuals as expected).

c. I think the risk of sample selection should be recognized. Both the kidney and the brain deteriorate with age. I wonder whether there could be collider or survival biases affecting the

analyses and how this could be prevented. Calibrating GWAS results to the reference population (least for the kidney part) might be a valuable sensitivity analysis to conduct (PMID: 40234401; PMID: 37106081)

Thank you. Our analyses are making use of large-scale summary-level data. The evaluation of collider or survival biases is a complex methodological question that is beyond the scope of this current work.

4. Suppl. Tab. 1 should be moved to the main manuscript (maybe including the information in Fig. 1 or as a separate table). Please, add the source of the GWAS.

Thank you. GWAS sources are presented in Table 1, and the previous Supplementary Table 1 with the same sizes is now a main table (Table 1).

5. Prior to entering the genetic correlation analyses, where GWAS summary statistics corrected for genomic inflation?

GWAS summary statistics were not corrected for genomic inflation prior to entering the genetic correlation analyses. We have calculated and added lambda GC to Supplementary Table 1. All results had a genomic control lambda close to 1 (indicating no inflation), with eGFR having slightly higher values (~1.4). Given that eGFR is a highly heritable polygenic trait (i.e. hundreds of loci across the genome are associated with eGFR), such a genomic control lambda is expected, but we note that the LD score regression intercept (which distinguishes between confounding vs. polygenicity and is also presented in Supplementary Table 1) is very close to 1 across all traits, including eGFR. We have added the following text to the Results section to note these observations: "SNP-based heritability estimates and LDSC intercepts for each trait are reported in Supplementary Table 1. LDSC intercepts were close to 1 across all traits (range: 0.90-1.08), indicating minimal confounding from population stratification or other biases."

6. Given the work presents many levels of analysis (with/without proxy cases; sex-stratification), I would recommend to avoid running both strict and lenient gene-set enrichment analysis with LAVA: please, limit to "strict".

We now only present strict gene-set enrichment results. Additionally, following the LAVA developers' recommendation to use UK Biobank rather than 1000 Genomes as the LD reference for European ancestry analyses, we reran all LAVA analyses accordingly; no results passed multiple testing correction.

7. Line 352: please, avoid using "etc." when presenting results.

We have removed etc.

8. Line 359: a "." is missing after the parenthesis
The period has been added after the parenthesis.

Reviewer #2

Gawhary et al. present global and local genetic correlation analyses between kidney-related traits and late-onset neurodegenerative diseases (Alzheimer's disease and Parkinson's disease). They collected large-scale publicly available summary statistics, with and without inclusion of proxy cases or sex-stratification. While statistical power for AD is limited, they found several significant global genetic correlations between kidney-related traits and PD. Local genetic correlation, gene-set enrichment, and network-based enrichment analyses were conducted, but limited mechanistic insights are provided beyond the number of significant results.
While the manuscript is of interest and could be one of the first studies to systematically examine genetic correlations between kidney-related traits and neurodegenerative diseases, there are a couple of major concerns.
Thank you for your review. We have outlined below how we have worked to address them so to enhance interpretability and relevance.

1. Local genetic correlation results severely lack interpretability. One of the major advantages of local genetic correlation analysis over global genetic correlation is that it allows investigation of which genetic variants contribute to shared genetic architecture between traits. Currently, the manuscript only presents the numbers and fractions of overlaps, and buries the majority of the results in numerous Supplementary Tables or Figures. To mechanistically interpret the shared local genetic architecture between kidney-related traits and neurodegenerative disorders, the authors should provide a detailed investigation of why two traits show significant local genetic correlation.
We have now significantly streamlined the manuscript and supplement so not to intentionally dilute the investigation of the potential underlying biology with the methodological details. In particular, we have substantially improved the interpretability of the local genetic architecture results. While no LAVA results passed multiple testing correction in the revised analyses (using the UK Biobank rather than 1000 Genomes reference as now recommended by the LAVA developers), we have added conjFDR analysis as a complementary locus-level approach, followed by Bayesian colocalization to distinguish shared from distinct causal variants at each locus, and eQTL annotation to link colocalized variants to their likely target genes. This multi-layered approach — conjFDR → colocalization → eQTL — provides the mechanistic interpretability that was previously lacking.

2. Similarly, only limited results are provided for gene-set enrichment or network-based enrichment analyses in the main text. Only a few loci, such as NR1H2/NR1H3 for AD and

metabolism, are documented in the text, with limited description of their potential implications or relevance.

Thank you. We now better balance the amount of space dedicated to each set of results, by increasing the description of the enrichment Results section to include specific pathway names, FDR-adjusted P-values, and discussion of which traits drive which enrichment signals. The Discussion now explicitly connects the enrichment results to the colocalization and eQTL findings, showing convergence on the same biological theme from two independent analytical approaches.

3. Main figures only cover global genetic correlation analyses. It is disappointing to see that the figures are only heatmaps of global genetic correlation and that none of the local genetic correlation, gene-set enrichment, or network-based enrichment analyses are represented in the main figures.
We have completely reenvisioned the main figures. We now have 3 main figures to: (i) visually summarize our study, and to display two key sets of results: (ii) the global genetic correlation results, and (iii) the colocalization results.

Reviewer #3

This study investigates genetic correlations between six kidney-related traits and two late-onset neurodegenerative diseases (Alzheimer's and Parkinson's). Using GWAS data, the authors estimate both global and local genetic correlations, analyzing the influence of proxy cases and sex-stratification. Significant correlations were identified for Parkinson's disease with urinary sodium (Rg=-0.17) and urinary creatinine (Rg=-0.16) levels. Enrichment analyses point to shared immune, metabolic, and viral response pathways. The findings support a kidney-brain axis and emphasize the importance of considering sex differences and proxy inclusion in genetic studies. The analytical rigor, stratification by sex, and attention to proxy inclusion are commendable. However, important limitations in phenotype definition, particularly for urinary biomarkers, require major revision, as they impact the interpretation of the observed associations.
We thank the reviewer for the careful review of our work.

The study identifies significant genetic correlations between Parkinson's disease and urinary sodium and urinary creatinine. However, the lack of correction for urine dilution (e.g., using urinary creatinine as a denominator or normalization to osmolarity) introduces major interpretative uncertainty. Urinary sodium in spot samples is highly sensitive to hydration status and recent dietary salt intake, and without normalization (e.g., uNa/uCr), may reflect extrarenal factors rather than kidney-specific function. Urinary creatinine itself is a function of muscle mass, hydration, and tubular handling, complicating its interpretation as a standalone phenotype. Lastly,

diuretic use, common in aging populations and in PD in particular (due to comorbidities like hypertension), can dramatically alter urinary sodium levels.

Given these limitations, the observed genetic correlations may reflect shared genetic influences on systemic volume regulation, salt handling, or hydration state, rather than intrinsic renal function per se.

To strengthen the biological interpretation of the findings, it is necessary to recompute key urinary phenotypes with dilution correction, ideally as ratios to urinary creatinine (e.g., uNa/uCr or UACR). It would further help to exclude or stratify by diuretic use. Such refinements would help disentangle kidney-specific effects from broader homeostatic or external influences, and provide a more reliable readout of kidney–brain genetic correlation.

Thank you. Reviewer 1 had a similar comment. We have now re-performed analyses using generally accepted indicators of kidney function: creatinine-based eGFR (estimation of glomerular filtration), uACR (measure of protein in the urine) and hematuria (measure of blood in the urine) as well as urinary sodium and urinary potassium as ratios to urinary creatinine.

In addition, for phenotypes showing significant local genetic correlation, colocalization analyses could help clarify whether observed overlaps are driven by shared causal variants. This would complement the LAVA results and provide further insight into potential mechanistic links between kidney traits and neurodegeneration.

We agree, and we have implemented colocalization analyses that go substantially beyond what was suggested. Specifically:

We used coloc.susie rather than coloc.abf, integrating Sum of Single Effects (SuSiE) fine-mapping to accommodate multiple causal variants per locus. This is now considered the recommended approach for colocalization, as coloc.abf assumes a single causal variant per locus, an assumption that can produce unreliable results at complex loci.

We also performed eQTL annotation of colocalized variants using GTEx v11 single-tissue cis-eQTL data in brain and kidney tissues.

These additions substantially enhance the mechanistic interpretability of our findings, as the reviewer recommended. The colocalization results are now presented in both the Results and Discussion, and visualized in the new Figure 3.

In summary, this is a well-designed and timely study with high potential impact. To substantially improve the validity of the results, it is essential to address urinary dilution correction. Furthermore, we recommend extending the analysis with colocalization studies to enhance interpretability and translational value.

We appreciate this feedback. As noted above, we have now only focused on traits with clear kidney implications (eGFR, uACR and hematuria) to improve validity of the results, incorporated colocalization analyses to enhance interpretability.

We thank the reviewers for their careful assessment of our revised manuscript. We outline our responses to the individual comments in bold below. For your reference, we include a tracked-changes version of the manuscript as well as a clean version.

Reviewer #1 (Remarks to the Author):

I'd like to thank the Authors for their revision of the manuscript. However, my main concerns remained only partially addressed. In my previous report, I mentioned several potential risks of confounding. The most important one is that creatinine-based eGFR partly reflects muscle mass metabolism. Muscle mass changes with age, along with the risk of PD. For instance, cellular senescence might affect both PD risk and creatinine levels through muscle mass changes, without necessarily going through kidney dysfunction. At minimum, this risk should be acknowledged in the Discussion. In addition, GWAS of cystatin C do exist and I think Authors could attempt a sensitivity analysis (to avoid disrupting the current flow of presentation).

Thank you for reviewing the revised version of our manuscript and for noting these concerns. We have now addressed this important point in two ways. First, we conducted a sensitivity analysis using cystatin C–based eGFR (eGFRcys; n = 460,826; Stanzick et al., 2021), a filtration marker largely independent of muscle mass. In particular, we have tested PD-cystatin-C-based-eGFR through the identical pipeline (conjFDR through to coloc.abf) (Supplementary Methods; Supplementary Results; Supplementary Tables 12 and 18). We observed that the key shared signal from the PD-creatinine-based-eGFR remained: the *BIN3* locus colocalized with eGFRcys (PP.H4 = 0.87; candidate shared variant rs13264187), matching its colocalization with creatinine-based eGFR, while the PD risk locus *SCARB2/FAM47E-STBD1* remained distinct (PP.H3 = 0.9999). Because cystatin C is largely independent of muscle mass, identification of the *BIN3* colocalization suggests that this shared PD–kidney signal is unlikely an artefact of the muscle-mass component of creatinine. We note that eGFRcys (n = 460,826) is derived predominantly (~95%) from UK Biobank (n = 436,765; within-cohort linear mixed model), with a ~5% contribution from the CKDGen European meta-analysis (n = 24,061; Stanzick et al., 2021).

Second, we have now added an explicit acknowledgement to the Discussion that creatinine-based eGFR partly reflects skeletal muscle mass, and that processes such as sarcopenia and cellular senescence could in principle influence both PD risk and creatinine independently of glomerular filtration: "[...] creatinine-based eGFR estimation may partially reflect muscle mass metabolism in addition to kidney function. We therefore conducted a sensitivity analysis using cystatin C-based eGFR,

which is largely independent of muscle mass and in which the primary shared locus (*BIN3*) was recovered (Supplementary Material).”

Where this is addressed in the revised manuscript: Discussion (limitations paragraph); Supplementary Methods 9, “Sensitivity analysis using cystatin C-based eGFR,” and the accompanying Supplementary Results; Supplementary Tables 12 and 18.

Reviewer #2 (Remarks to the Author):

I thank the authors for their extensive revision. In response to the review panel, they have narrowed the scope to PD and revised their methodological approach, adopting conjFDR and colocalization with SuSiE fine-mapping in place of LAVA. My second and third comments from the first round are largely addressed.

While the manuscript is now more focused and the analytical toolkit appears more modern, I have several major concerns regarding the revised approach and its interpretation.
Thank you for reviewing our manuscript. We outline our responses to your individual comments below.

1. Colocalization and SuSiE fine-mapping of meta-analysis GWAS with an external LD reference panel are not reliable.

In the revised pipeline, the authors use coloc.susie (Wallace et al. 2021) to perform colocalization with SuSiE fine-mapping. This approach has serious methodological flaws.

a) Multiple-causal-variant fine-mapping with SuSiE requires accurate in-sample LD. Substituting an external LD reference (1000 Genomes Phase 3 EUR, with only ~500 samples) is strictly not recommended and produces many false positives (Ulirsch et al. 2019; Weissbrod et al. 2020).

b) Meta-analysis makes this worse. Both input GWAS are themselves meta-analyses: the sex-combined PD sumstats are a meta-analysis of the sex-stratified datasets, and the sex-combined eGFR sumstats are a meta-analysis of CKDGen and UKB. This is precisely the setting in which the 61-locus PD-eGFR signal and all ten "shared" colocalized loci arise. Beyond the absence of in-sample LD, fine-mapping and colocalization of meta-analysis

sumstats are known to be miscalibrated and error-prone (Kanai et al. 2022).

These issues are the most likely reason SuSiE failed to converge at the majority of loci (only 10 converged). The coloc.abf fallback does not resolve the problem, because it relies on single-causal-variant ABF fine-mapping, and the meta-analysis miscalibration still applies.

Given these severe flaws, the fine-mapping and colocalization results presented in this study cannot be considered reliable.

We thank the reviewer for highlighting these significant flaws in the coloc.susie approach. This critique led us to revise the colocalization analysis using approach not requiring an LD panel, and we also assessed the impact of meta-analysis.

(a) We no longer use an LD reference panel in colocalization analysis. We have replaced the SuSiE-based colocalization with an evaluation at each locus of interest with coloc.abf (Giambartolomei et al., 2014; coloc v5.2.3), which computes colocalization posteriors from the marginal association statistics under a single-causal-variant assumption and takes no LD matrix as input. The external-LD concerns of point (a) — the 1000 Genomes reference and the SuSiE convergence failures — therefore have been mitigated through the use of coloc.abf for colocalization.

Specifically, colocalization was performed at all non-MHC conjFDR loci across the seven PD–kidney trait pairs (128 locus–trait-pair tests; Supplementary Table 12) using coloc.abf, with conservative priors set to increase confidence in the findings (p1 = p2 = 1×10^{-4}; p12 = 5×10^{-6}), and a shared signal defined as PP.H4 > 0.80.

(b) We thank the Reviewer for pressing on this point, and we completely agree that fine-mapping and colocalization of meta-analysis summary statistics can be miscalibrated. We do not claim that either input GWAS is exempt from this — the PD statistics are a meta-analysis across the IPDGC datasets and UK Biobank (Blauwendraat et al., 2021), and the eGFRcrea statistics a meta-analysis of CKDGen and UK Biobank. We have now tried to address this concern from several complementary angles (at the level of inference, through replication, and empirically with the Reviewer's suggested diagnostic) which we hope provide evidence that our presented results are not primarily driven by miscalibration.

First, we want to acknowledge that this is a legitimate and important concern, and we have been careful not to over-interpret our results in light of it. Our conclusions are not intended to rest on identifying the specific causal variant (as coloc.abf asks only

whether a locus is better explained by a single shared causal variant (H4) or by two distinct causal variants (H3)), and our conclusions do not attempt to pinpoint the exact causal variant. Because the miscalibration Kanai et al. describe bears most directly on single-variant fine-mapping (that is to say, the assignment of posterior probabilities to individual variants), we would tentatively suggest that its effect on the broader shared-versus-distinct judgement we report is likely to be more limited. To be cautious, we have ensured that our conclusions are at the shared-versus-distinct level, and we have treated each eQTL-based gene assignment simply as a candidate rather than a definitive assignment.

This shared-versus-distinct inference is furthermore stable across a grid of colocalization priors — the evidence favours a shared over a distinct causal variant at every locus and prior (Supplementary Table 20) — and the shared PD–eGFR loci persist under stricter conjFDR thresholds (Supplementary Table 19).

Second, we replicated the core signal – the colocalization at *BIN3* - in a different, lower-powered biomarker whose construction largely sidesteps the cross-cohort heterogeneity at issue: cystatin C–based eGFR (eGFRcys; n = 460,826), derived predominantly (~95%) from a single cohort (UK Biobank, n = 436,765; within-cohort linear mixed model, with ~5% from the CKDGen European meta-analysis, n = 24,061). In this analysis, the BIN3 colocalization is recovered (PP.H4 = 0.87) and *SCARB2/FAM47E-STBD1* remains distinct, indicating that the strongest shared signal unliekly an artefact of cross-cohort meta-analysis.

Third, we screened directly for the specific artefact that the Reviewer's framework targets. We applied SLALOM (Kanai et al., 2022) — which flags loci suspicious for meta-analysis fine-mapping by detecting association-statistic outliers against a local LD reference via a simplified DENTIST test (Chen et al., 2021) — to every reported colocalized locus and the *SCARB2/FAM47E-STBD1* distinct-variant control, for both traits, against a large in-sample UK Biobank LD reference (N = 337,462 unrelated white-British individuals) used, following Kanai et al., solely to diagnose summary-statistic/LD inconsistency and not for fine-mapping.

Across all reported colocalized loci (18 locus–trait tests) SLALOM identified no DENTIST-S outlier variants (see the newly added Supplementary Table 21), and the distinct-variant control behaved as expected. Because SLALOM screens for a specific inconsistency rather than certifying posterior calibration, and because our array-based in-sample panel is best powered where the lead variant has many LD partners (*BIN3*, 26 variants) and thinner where few variants exceed $r^2 > 0.6$ with the lead (e.g., *RAB19*, *TNK2*), we present it as a corroborating screen — clean at the best-powered

loci — that supports, rather than supplants, the resolution-level and replication evidence above.

Under this reference-free, conservative analysis, of the 70 distinct non-MHC loci, four show a shared causal variant (PP.H4 > 0.80), a fifth meets a composite criterion (PP.H3 + PP.H4 ≥ 0.8 and PP.H4/PP.H3 ≥ 5), and eleven show distinct causal variants (PP.H3 > 0.80); the remainder are inconclusive.

Notably, the two loci that had reached PP.H4 = 1.00 under the previous external-LD SuSiE analysis — the over-confident pattern external-LD fine-mapping can manufacture — were re-evaluated: the chromosome-1 *ZBTB7B/DPM3* region now resolves to distinct causal variants (PP.H3 = 1.00), and the chromosome-8 *MSRA/PRSS51* region is recovered at a conservative posterior (PP.H4 = 0.93). The Methods and Results have been updated accordingly.

Where this is addressed in the revised manuscript: Methods, "Colocalization analysis" (now reference-free coloc.abf, no LD reference panel); Results, "Colocalization and eQTL annotation"; Discussion (colocalization paragraph); Figures 1 and 3 (workflow schematic and legend updated to coloc.abf); Supplementary Methods 6 (Bayesian colocalization), 9 (eGFRcys replication), 10 (prior sensitivity), and 11 (SLALOM diagnostic); Supplementary Tables 12, 19, 20, and 21.

2. Even taking the colocalization results at face value, the central "shared architecture" framing is not well supported.

Across 128 non-MHC loci, distinct causal variants (PP.H3 > 0.8, n = 40) outnumber shared causal variants (PP.H4 > 0.8, n = 10). The dominant empirical result is therefore that PD and kidney-trait signals colocalize to the same regions but are driven by distinct variants. The Discussion acknowledges this, yet the title, abstract, and summary statements continue to lead with "shared genetic architecture." I ask the authors to recalibrate the framing throughout to reflect the predominance of distinct causal variants, reserving the shared-variant interpretation for the minority of loci, concentrated in PD-eGFR, that genuinely meet the colocalization criteria.

Thank you. We agree and have recalibrated the framing throughout — not only the title but the abstract, summary statement, and Discussion.

We have revised the title to "Genome-wide cross-trait analysis to compare genetic architecture between Parkinson's disease and kidney-related traits," removing the "shared architecture" emphasis.

The lead message of the title, abstract, and summary is now that PD and kidney-trait associations converge on the same loci but are typically driven by distinct causal variants; the shared-causal-variant interpretation is reserved for the minority of loci, concentrated in PD–eGFR (*RAB19, MSRA/PRSS51, BIN3, TNK2*), that meet our criteria.

Where this is addressed in the revised manuscript: Title; Abstract; Results, "Colocalization and eQTL annotation"; Discussion (colocalization paragraph); Supplementary Table 12.

3. The robustness of the conjFDR results should be established.

The original manuscript's central claims rested on LAVA local genetic correlations. Once these became non-significant under the recommended UK Biobank LD reference panel, the authors replaced LAVA with conjFDR as the primary locus-discovery method, without explicitly justifying this substitution. Because the revised LAVA analysis is now completely null, the new conjFDR results require independent support to reconcile these apparently contradictory findings.

The number of shared loci tracks the statistical power of the kidney GWAS: PD-eGFR yields 61 loci, whereas the smaller kidney GWAS yield between 0 and 11. Because conjFDR < 0.05 is a permissive criterion and is sensitive to differential power between the two input datasets, the possibility that the PD-eGFR signal is inflated by the exceptional power of the eGFR GWAS should be addressed directly, for example through threshold-sensitivity analysis or a down-sampling or power-matched comparison.

Thank you. The conjFDR approach is not a substitution for LAVA but is a complementary approach to interrogate potential shared genetics. ConjFDR asks whether individual genetic variants are pleiotropic (i.e. shared) between the pair of traits, whereas LAVA asks whether the genetic variants at a locus of interest show evidence of correlation between the pair of tested traits.

We now more clearly justify our use of conjFDR explicitly and reconcile it with the LAVA result. LAVA and conjFDR are not interchangeable: LAVA estimates the local genetic correlation between two traits within predefined LD blocks — a signed, region-averaged quantity that requires detectable local heritability for both traits and a consistent direction of effect across the block — whereas conjFDR identifies individual loci jointly associated with both traits and is agnostic to the direction of effect (Smeland et al. 2020). The conjunctional FDR is the maximum of the two conditional FDRs and is therefore an upwardly biased, conservative estimate of the

true FDR; conjFDR < 0.05 is the established convention in this framework (Andreassen et al. 2013; Smeland et al. 2020) — that is, conjFDR is conservative rather than permissive. Given that our objective was to identify specific shared loci, conjFDR is appropriate as a primary analysis: "LAVA and conjFDR address complementary questions. LAVA estimates the direction-dependent local genetic correlation within LD blocks, whereas conjFDR identifies discrete loci jointly associated with both traits independent of effect direction. As our aim was to identify specific shared loci, we used conjFDR as the primary locus-discovery method."

The two findings are concordant rather than contradictory. We observe weak global genetic correlation (significant only for PD–uK/Cr) and null local correlation (LAVA) alongside numerous discrete conjFDR loci — the signature of genetic sharing concentrated at specific loci with heterogeneous effect directions rather than diffuse polygenic overlap. This is precisely what we observe at the variant level: PD risk alleles are associated with higher eGFR at some loci (*TMEM163, BIN3*) and lower eGFR at others (*FAM47E-STBD1, RAB19*). Such mixed directions average to a near-zero genetic correlation, rendering both global rg and local LAVA null, while conjFDR — being direction-agnostic — still detects each locus. The additional stringency of LAVA (a per-block univariate heritability filter for both traits, Bonferroni-corrected across 2,495 blocks) further reduces its power.

To address directly the concern that the PD–eGFR signal might be inflated by the exceptional power of the eGFR GWAS, we performed a threshold-sensitivity analysis: the number of independent non-MHC PD–eGFR loci falls from 58 at conjFDR < 0.05 to 16 at < 0.01 to 8 at < 0.001, and the core loci — *FAM47E-STBD1/SCARB2, CTSB*, and *TMEM163* — persist at the strictest threshold (Supplementary Table 19). Because conjFDR controls the FDR conservatively, additional loci at higher power reflect additional true discoveries rather than inflation; the guard against false "shared" claims is the colocalization step (at which distinct variants outnumber shared), not the conjFDR count. The conjFDR loci are further supported by our downstream analyses — reference-free colocalization and cis-eQTL annotation corroborate specific effector loci (*BIN3, MSRA, TNK2*, and the kidney effector *CCAR2*) — and the lower-powered eGFRcys analysis (n = 460,826, versus 1,201,930 for creatinine-based eGFR) recovers the core shared signal (*BIN3* colocalized, PP.H4 = 0.87). We now state this rationale in the Methods.

Where this is addressed in the revised manuscript: Methods, "Identification of shared genomic loci" (explicit conjFDR rationale); Discussion (reconciliation of the conjFDR loci with the null LAVA result); Supplementary Methods 5 and 12, "conjFDR threshold

sensitivity”; Supplementary Table 19.

Reviewer #3 (Remarks to the Author):

Thank you very much for addressing my questions and concerns. I believe the revisions have substantially strengthened the manuscript.
Thank you for reviewing our manuscript.

We thank the reviewers for their careful assessment of our revised manuscript. We outline our responses to the individual comments in bold below. For your reference, we include a tracked-changes version of the manuscript as well as a clean version.

Reviewer #1 (Remarks to the Author):

I'd like to thank the Authors for addressing my concerns. I have no further questions.

Thank you for reviewing our manuscript.

Reviewer #2 (Remarks to the Author):

I thank the authors for their extensive revision. The revised manuscript addressed the majority of my concerns, including the revised colocalization methods and the recalibrated title and abstract.

Thank you.

I only have one minor comment. While the authors claimed the colocalizations "were stable across a grid of priors", ST20 showed PP.H4 <= 0.80 at p12 = 1e-6 (stringent) for four of the five shared calls. Minor limitation texts would suffice.

We thank the reviewer for catching this. They are correct that at p12 = 1 × 10^{-6}, four of the five shared-variant calls fall below PP.H4 = 0.80 (0.37-0.72), and only *RAB19* remains above it (0.85 sex-combined; 0.83 male-specific).

We have corrected the sentence in the Results and we have added a limitation to the Discussion, as suggested.

Results, Colocalization and eQTL annotation:

These colocalization calls held across the grid of priors tested down to p12 = 5 × 10^{-6}; at the most stringent setting (p12 = 1 × 10^{-6}) four of the five shared-variant calls fell below

the 0.80 threshold, although PP.H4 continued to exceed PP.H3 at every locus (Supplementary Table 20). None of the calls were flagged for meta-analysis fine-mapping miscalibration by the SLALOM diagnostic (Supplementary Table 21).

Discussion, limitations:

Fifth, colocalization posteriors depend on p12, the prior probability that a variant is causal for both traits. Our shared-variant calls hold across the priors in common use, but only RAB19 remains above 0.80 at p12 = 1×10^{-6}. The evidence at MSRA/PRSS51, BIN3 and TNK2 is therefore conditional on standard prior settings, although at no prior does it favor a distinct causal variant.
